# Extra-Limites

**Published:** 1841-07-01

**Authors:** 


					1841]
( 289 )
EXTRA-LIMITES.
0
-????}-?>*?-
Notes on the Medical Topography of Alston Moor. By
W. Ewart, Esq.
Tiie
Upon Vlt^ ex^stence of man, as well as that of all other organized beings, is acted
?f j an(l variously modified by surrounding influences?life being an assemblage
WclleTmena dependent not ?nly on certain conditions belonging to the object
is pi 1 displays it, but on other states, likewise, of the locality where that object
In ' ^ must abvays be characterized by their conjoint peculiarities.
c?nsirl ^ing t^ie Physiol?gy and pathology of organization, the elements for
(Jes ?rati?n are numerous and diversified; and none amongst these are more
tudeg ?? attention than the cosmical and social. Man in his growth, habi-
as Suls health and disease, bears an evident impress of exterior agency?and
tyy.0>. > the influences of climate, social customs, and industrial pursuits, are every
be traced.
a tn.0,st every country of the globe in its aggregate of human disease, manifests
SaQie?V a c^sease which is never seen amongst the people of another, whilst the
fro disease, should it happen to be endemic in both, often derives a modification
as m s9Ine local agency. The causes of disease therefore, and their modifications
theirani *n vai'i?us situations, will require the medical enquirer, who attempts
cap , ?tudy, to direct his eye perseveringly upon every element possessed by these
a rat influencing in the least degree organic actions. And thus alone can
V ?nal doctrine of hygiene applicable to each particular place be founded.
aSp t ?r)ly do the well-defined climatic divisions of the earth produce peculiar
th0uCrf disease, but the different localities likewise of the same country, even
this r seParated by a few miles only, often manifest the same character?and
phvs* lv,ersity will be found to be almost wholly assignable to some difference in
ral f an(l social particulars. Look for instance at the dissimilarity in the gene-
So jCatures disease to be met with among the cotton mills of Lancashire?
attiorftrUmental *n the development of nervous excitability?to those found
Plain Vascular agriculturists. The latter again, residing in fertile valleys, or
tain S rich in vegetation, are often exposed to causes of disease which the moun-
ai0 er never meets with whilst he breathes the pure air of heaven as it sweeps
Ca& the bleak mountain side, which exhales no miasmata, and where contagion
inherit 's not ^ without his share of the many diseases which man
r|"*r
te ? study of medical topography is far from being uninteresting, not only as
stiti t- *ts hearings upon the prevention and treatment of disease, but as con-
Scuri lng an important branch likewise of natural history. Though much ob-
by as yet hangs over the essential nature of vital phenomena, and the laws
eftal 11 ^ey are subject to modification by external agency, yet we are often
tnat ^ to trace clearly the effects of meteorological operation, and that of other
au^ ria| things, though their modus operandi may remain a mystery?thus heat
t^em C?i bought and moisture, geological and botanic character, often force
acti0 ves upon the attention as evidently implicated in the modifying of vital
Al>f' an^ the production of disease.
Hjjp s on-m?or, famous for several hundred years for the produce of its lead
of j)S' j"orms the eastern extremity of Cumberland, where it joins the counties
ranepU^ham and Northumberland; it constitutes a part of that bleak mountain
is peculiar to itself.?Thus the inhabitants of one nation are the frequent
S0ltthe?f 1^00rlan(l' called the " Penine Chain," which stretches far away in a
\t r X direction from the borders of Scotland. Cropfell, the highest elevation
^?LXIX. U
290 Extra-Limites. [July ^
of that range, bounds it on tlie West, from whence it stretches for some miles jjj
the East, in a series of undulating hleak barren fells, at the bases of which' ,
villages, or isolated cottages, are scattered the abodes of the lead miners?situa
as it is, in a part of the island of no great breadth between the German Oc?jaj
and Irish Sea, and at a considerable height?perhaps above any other inhaW
place in England?its climate is characterized by coldness and moisture, and )
frequent and sudden alternations. Changes of temperature are particularly ? g
quent and striking. In the course of twenty-four hours these may often ^
witnessed to supervene in the most abrupt manner?intense frosts succeeding
dripping thaws, or vice versa. Its surface is almost altogether unprotected v]
plantations, allowing the winds to sweep over it unbridled, and its valleys a
open and thoroughly ventilated.
On a general view of the diseases which prevail in Alston-Moor, it is instant'}
evident that it enjoys an eminent freedom from fevers of contagious cliaracter'
These seldom indeed originate amongst its inhabitants, and when they
brought by some infected person from any other situation, they generally cj'e
with the death of their bearer, or disappear with his recovery.?Once during
past year have typhus fever and small-pox been respectively imported from a d's'
tance, but they immediately became extinct, and without any particular prec?U"
tions being had recourse to for the purpose of effecting such a result?as if
climate of Alston-Moor was fatal to their prevalence. In the same way,
sporadic cases of the kind do occur, they show almost no tendency to spread'
This immunity from contagious disease, and hostility to its propagation, may
perhaps in some degree be connected with the physical character of the country-
The atmosphere seldom stagnates upon it, and the suiface of its "fells" are f?r
the most part covered with moss, the antiseptic qualities of which, in its cofl'
tinned carbonization, may have some effect in preserving the air free froma
malarious constitution. This the writer, at least, in his limited experience, haS
sometimes observed, that people residing upon tracts of mossy countiy are sing11'
larly free from diseases of virulent contagious type. The effect here conjecture'1
however requires the confirmation of more extensive observation.
The diseases which prevail most extensively in Alston-Moor are the inflam-
matory and neuralgic, though the former very much preponderate. During
very severe winter season in particular, inflammation, or diseases approaching t0
it in nature, affecting the dermoid, mucous, glandular and fibrous textures, are
exceedingly abundant. Thus some one or other of such diseases as ophthalmia
tonsillitis, tracheitis, bronchitis, diarrhoea and rheumatism, with exanthemata13
and papular or pustular eruptions, are almost daily to be met with. Pulmonary
tubercle too forms an important item in the aggregate of disease, and the clima^
seems to lend awful facilities to the progress of those processes, by which,111
defiance of all medicine, it hurries its devoted victims to their graves. Paren-
chymatous inflammations likewise, such as cerebritis, pneumonia, hepatitis, &c- aS
in all other situations, are sometimes seen, as are also fevers of the remittent type
amongst the younger class of people, but these form a trifling number in com-
parison with the former. Inflammation of the genito-urinary mucous membran^
particularly that species, the prime prophylactic of which is a sound morality*lS
by far the most scarce of all.
There are two diseases, which, though not exclusively confined to Alston-
Moor, yet as evidently depending on local influences, may be perhaps worth 3
short consideration. These are the miners' bronchitis?in local phraseology
" grovers complaint"?and bronchocele. The former is a disease which sooner or
later, and in one form or other, attacks almost all lead miners. It assumeS
various aspects according to the simplicity or complication in which it may
manifested. Its cause is the constant inhalation of minute irritating dust pr?"
duced by blasting the various argillaceous, siliceous, or calcareous stratification*-
through which the miner must work his way in his search for ore, and in work'
1841]
Medical Topography of Alston-Moor. 291
Parti ?re w^en f?und- This breathing of an atmosphere loaded with irritating
thorny?8 seems scarcely ever to be the exciting cause of acute inflammation,
Ijeij, 'j1 highly predisposes to it?the continued congestion produced by it,
citino-eas1^ developed into active inflammation by exposure to the common ex-
moi? Causes. And when this has been once effected, it is too apt to continue
ye e or less in a chronic form. But even though the miner should for many
?r e'S yfCfI)e an attack of acute bronchitis, the air which he daily respires for six
tant ? urs> loaded as it is in various degrees with impalpable physical irri-
int S'Wlh for the most part gradually produce a chronic bronchitis of a very
actable kind.
es -ost all lead miners, after having pursued their employment for some time,
are - *n a badly ventilated mine, though in other respects in perfect health,
inst?a an^ exertion affected with dyspnoea. On quickening their pace for
stru 01 ascen(iing an eminence, their breathing becomes hurried and ob-
Cou c ec*- This evidently arises from the congested state of the bronchial mu-
are S rneinbi'anc, and the secretion which it exudes, abnormal quantities of which
in tfPeCt0rated' generall>r tinged of a bluish cast by the powder that is inhaled
hai 6 m'ne" The miner may continue in this state for many years, subject per-
PS to occasional slight aggravations from catarrhal attacks, but able without
cil inconvenience to pursue his avocations. At last, however, his cough be-
es gradually more frequent and teazing. His appetite fails, especially in the
te rning> he frequently leaves his breakfast untouched, and sets out for the sub-
(],.tanean scene of his operations with feelings of languor and nausea. His
>spnoea is more urgent, which, with increasing debility, obliges him to rest
Pjstedly in walking even the distance of three quarters of a mile. His ex-
?toration increases in quantity, and for the most part consists of frothy and
d !. rin mucus, sometimes streaked with blood. His strength has now much
Pli i e.^' is much emaciated, and his partially suffused countenance and pur-
ov 1 tln?ed hps indicate obstruction of the pulmonary circulation and mal-
sey '?nati?n of the blood. He is now unable to work, and the increasing
?er*y of his ailments confines him to his house. His cough gets worse and
are and there is more and more expectoration. Sonorous and mucous ronchi
re .Very evident to the most inexperienced auscultator; but the several pulmonic
dehT*8 ?ound generally clear enough on percussion. Hectic fever supervenes,
e * ty increases, and from a few weeks to some months after relinquishing his
. Payment, he sinks exhausted. Such is one form of bronchitic affection dis-
ced by the lead miners of Alston-Moor.
as l1' ^ie miner after feeling a gradual impairment of health for some years,
debTdy descrihed, and continuing his employment, though from dyspnoea and
br ty very unfit to do so, is while still working suddenly seized with acute
asph -t*8' I)ei"haps conjoined with pneumonia, and in two or three days droops
aren ?t^er cases, the miners, while still comparatively young, healthy, and able,
Co Seized with acute bronchitis. By appropriate treatment this is sometimes
plo P ly subdued, allowing those who had been affected to return to their em-
,y yment, but at other times, in spite of all remedies, it lapses into a chronic form.
hritative fever continues to prey upon the system, the cough is very
pe,.t some, and great quantities of bluish frothy and puriform mucus are ex-
few 0ra^ed? especially towards morning. The patient often dies exhausted in a
the ^Veeks or months. In two fatal cases of the latter kind which came under
-iter's observation, there were expectorated, towards the end of their course,
of fl earth)' concretions. In one patient two or three small bits about the size
teetlf^6116^ P^n's heads were ejected with the expectoration and grated upon his
irr * In the other, a much larger concretion, about the size of a pea, and of
loojfto shape, was coughed up, imbedded in a sputum, black and gelatinous
lngin the centre, which was surrounded with matter of puriform appearance.
U 2
292 Extra-Limites. [July ^
It \va8 of a white colour, and seemed to have been moulded into the shape
possessed, being pyriform, and having one or two small projections blackened ^
the top. On treating it with dilute muriatic acid it was dissolved, disengages
minute bubbles of gas, indicating it to be the carbonate of lime. In the solute
there floated small fiocculi, which appeared to be animal matter that had co
stituted part of its composition. These concretions were no doubt the result
a depraved secretion in the pulmonary tissue, and had the patients not been c
off so early as they were, it is probable that in time they would have been
subjects of manifest tuberculous disease. ^
There are various other aspects of chronic bronchitic disease evinced by |e' ?
. miners. Thus, some who have fortunately given up their mining occupati?. ^
before any serious structural derangement had been produced in their brond11^
apparatus, continue in after-life to display symptoms, more or less severe 1
various cases, of simple bronchial congestion, which seems of itself to have W ,
effect in abridging the term of their existence. In other cases an attack
acute bronchitis results in the establishment of a persisting irremoveable hro"
chorrhcea, characterized by dyspnoea, which has paroxysmal aggravations
lowed by a copious expectoration of a thin slightly viscid glairy mucus. A"
in one case, from the person's own account, has continued 15 years, since 0
exposure to cold he was seized with what, from the symptoms he describes a
having attended it, must have been acute bronchitis. In some cases the m1.11? '
after having laboured under bronchial congestion for some time, as produced ;
his employment, has a severe attack of hemoptysis, which leaves him much d ^
bilitated, and for the reminder of life he has dyspnoea accompanied with exCfL
sive irritability of the bronchial fibres, as evidenced by frequent attacks of a c"-,
convulsive whistling cough consequent on the least excitement. Sometifl>e?
bronchial congestion is accompanied by spasmodic asthma. In such cases tn _
usual dyspnoea is subject to sudden aggravations relievable by antispasmodics ^
warm coffee. Such is a very brief sketch of the various phases of the pulm0'
nary complaints to which the lead miners of Alston-Moor are subject.
diseases, I am given to understand, are much less prevalent now than they f?r'
merly were, which circumstance is ascribable to the improved ventilation of t&
mines, and to the praiseworthy exercise of their authority, by those who possesS
it, in the promotion of cleanliness and temperance amongst the miners.
As regards the treatment of these affections little need be said. In the
aggravated cases medicine avails but little in producing beneficial residts, but
others of more incipient character, and particularly when accompanied by gasti^*
hepatic derangement, much benefit may be derived from a proper course of me"1*
cine. In such cases a mercurial purge, followed by a few doses of a combination
of aloes, ext. hyosciam. and ant. tart, along with counter-irritation, have appear^
very effective in relieving functional oppression and the congested state of t*1
bronchial mucous membrane. The principal indication is, however, to rem?\
the miner from the operation of the irritating inhalations which prove either d1'
rectly or indirectly the cause of his complaints; but such can alone be done 0/
the relinquishment of his employment, which necessity perhaps compels him t0
avoid until it is too late.
Bronchocele exists rather extensively amongst the inhabitants of Alston*
Moor, which place has every claim to rank among the goitrous districts
England. It is principally to be seen affecting the female sex, at all ages fr0'11
seven or eight years upwards. Only two cases in the male have come under t"e
writer's notice, which however were, and still continue to be, tumors of no inco?"
siderable development. In both cases the morbid enlargement is principally ^
the lateral lobes, forming two large unseemly projecting tumors on each side o
the trachea. The connecting portion, though in some degree affected, is ?llCj
less so than the other parts. A considerable reduction of size has been effects
Medical Topography of Alston-Moor. 293
as vpf fcxt(rrnal and internal use of the iodine treatment, though much remains
fi U t0 acconiPlish-
Cele js t^s ^le roost interesting portion of the whole medical history of broncho-
excitino-lat re^at*n? t? 'ts etiology. Very much obscurity as yet hangs over its
search ? c^uses' f?r as the inquiry is surrounded with some difficulties, the re-
its elu68]- t^10se have directed attention to the subject, have done little for
f?r tl 1 Nothing can shew more clearly than the various causes assigned
his f 6 Pro^ucti?n of goitre, the limited nature of man's mental energies, and
than 1 ^lUent willingness to rest content with a plausible probability, rather
c0ln a ^mPt to push his way to the goal of truth through a rigid analysis, and a
ttiost en^Ve Auction of facts. As bronchocele occurs endemically for the
taino^art' ^ l?calities of similar physical character, viz. in the valleys of moun-
cause S rx?ions' ^ *s certain that these must in common contain its cause or
but th' ar'?us local peculiarities have at different times been assigned as such,
sive ()? exchisive morbid agency of which has been contradicted by more exten-
spr;nfr servation. Thus the use of melted snow, or the waters of calcareous
ceie. .s as a beverage, has by some been reputed the exciting cause of broncho-
liqui',1 J-lt supposition of such an effect, resulting from the use of these
entertS-ls *net by such a weight of antagonist testimony that it can no longer be
goitr aine(h That bronchocele may result from certain malaria possessed by
rnust?jUs districts is certainly not improbable, though if such is the case, that
r>a.? .e a kind very different from those other morbid agencies to which the
7UVS. generally applied.
\j0 glng from the historic details connected with the bronclioceles of Alston-
c0r r' a? given by the patients themselves, the inference is not unlikely to be
ag ct that, these thyroid enlargements are not the peculiar product of any one
t'oni exclusively> but that sevreral may concur in their development. On quet-
pjai o a considerable variety of affected persons as to the origin of their com-
Heck ,S' some confidently pronounce a cause, while others affirm that their swelled
tied S,Came gradually, and they know not how. A pretty large number of mar-
^ttril?0rnen ate the advent of their complaints from some difficult parturition,
their UtlnS their production to the straining of the parturient efforts, and state
SAveU'reaS?-n ^?r suPPosing such to be the case, is, that they first perceived the
AnotJ"- e'ther during a difficult labour, or immediately on its completion,
the o 'ei- c^ass persons, comprising both the married and unmarried, attribute
h'^1 their affections to their frequent employment in lifting heavy weights
the earing them upon the head. A few again blame some peculiar quality of
Co ater which they use?and it may be here stated that a great deal of the water
from y*1- *n Alston-Moor issues from the limestone?though the disease is far
har 1" confined to those who make use of such, nor do they shew any pecu-
sion la y to its supervention. Others, as already mentioned, make no preten-
Th CVen to a suPPosition, but freely confess their ignorance of the matter.
le two exciting causes most generally assigned, it will be perceived are one in
glanT'"61"' ^hey are evidently both such as to produce congestion in the thyroid
^ common with other parts of the head and neck; and being so, are not
form > 7 having their claims considered as to their effect in producing the
eh0cT s en^argement. They themselves certainly cannot be the cause of bron-
the c|-e' ^or.in other districts, where the inhabitants are equally exposed to them,
c0.0l1Sea?e *s never seen. We are consequently driven back to search for some
focal?).era^ng'. and more active cause, in the physical character of the goitrous
?ther ^ *-S eveiT way probable that the agencies already mentioned, as well as
all sif ?Perating in producing inordinate congestion in the thyroid gland, will in
the e Uatl.ons predispose to its enlargement. But as only some situations possess
deVeiSPec^ exciting causes, it will be in these alone, where the disease becomes
trjct ?P?o* Thus, what will assist in lighting up the disease in a goitrous dis-
5 will be altogether ineffectual for such a purpose in one of a different
294 Extra-Limites. [Juty ^
character. In the same way as that state of tlie system which in Egypt
subject its possessor to an attack of plague, will, in this country, have no sue
effect, where the immediate pestiferous cause exists not.
What seems to be the principal element that gives effect to all other co-opera'
ting agencies in the production of bronchocele in Alston-Moor, is its meteorolo'
gical character; in particular the frequent alternations of temperature so high1;
productive of vascular congestion. As has been already mentioned, these alter-
nations are in this locality particularly sudden and extreme?and if, by
means, such as straining, or these alternations themselves, the tone of the cap11*
laries of the thyroid gland, is in any degree diminished, its congestion is t0?
likely to be perpetuated by those meteorological causes to which the people
almost daily exposed, according to that law of the animal economy by wluc11
vasculkr determinations are generally directed to a weakened part. Considering
the peculiar structure of the thyroid gland, its loose yielding texture, and its m*
active unsecreting character, it is probable that it is little amenable to inflamn?3'
tion properly so called, and that those causes, which are generally so actuc
in exciting real inflammation and its train of consequences in other tissue^
such as the tonsils, parotids, and mucous membranes, in it only effect that
congestion too passive to be resolvable by purulent secretion, and there being 110
natural secretion to increase, it alone leads to that progressive hypertrophy whicl1
is the peculiar characteristic of bronchocele. It is well known that vascular con-
gestion is the grand cause of hypertrophy, whether in the overlabouring heart)
the blacksmith's biceps, or the urinary bladder straining to overcome obstruction
?the exalted energy of their functions only acting by attracting to them m?re
blood. It may be asked?why, then, if bronchocele is the product of the sain?
meteorological causes which so generally excite tonsillitis or catarrh, does it no1
likewise exist in those places where these diseases are often seen ? In answer,
may be be stated that, in no situation in Britain where the writer has as yf
seen, do these causes prevail so frequently, or with such intensity, as they do in
Alston-Moor. Neither has he ever witnessed the aforesaid inflammations so ex-
cessively abundant as they are in the place in question. And from this it
be inferred, that bronchocele is endemic only in those localities where alterna'
tions of temperature?or it may be other causes?productive of congestion an'1
inflammation, are in prevalency and greatness above the usual standard?and th<at
it is only one feature of their produce?one among the numerous class of con'
gestive and inflammatory ailments which will be found to obtain amongst a peo}>lc
exposed to these causes in such an exalted degree. Alternations of temperature
are not the only cause of local determinations, though they seem to be the grand
source of such in Alston-Moor : and being so, hold forth a strong claim to be
the principal exciting cause of bronchocele. Whether this affection is associated
with the same prevalency of congestive and inflammatory diseases in other
goitrous districts, the writer is unable to say. Should it be so, the views n?^'
stated will be strengthened?but if it is otherwise the circumstance will certainly
go far to invalidate them.
1841]
Treatment of Chronic Pleurisy. 295
^0TEs ?*v the Treatment of Chronic Pleurisy, with Effusion.
By the late James Hope, M.D.
for K ^^owing paper must possess a high interest for our readers, when we in-
Co'n, jem that it was commenced by Dr. Hope during his last illness, and
0 U('ed on his death bed. Unable to complete it as he could have wished, he
dav c,?mPeUe(l to dictate it in the shape of notes, which he finished only four
and h f^ecease- those who knew Dr. Hope well, this zeal for science
clia fellow-creatures will occasion no surprise. It was consistent with the
g0ojacter the man, who died as he had lived, an accomplished physician and a
rPVi
of tV|6 s^mPtoms ?f chronic pleurisy, with effusion more or less filling one side
(Cv ^ ?^les^ are perfectly well described by systematic writers, as Dr. Law,
ally ^ract- Med. ji. 395 a.) yet there is no class of affections more habitu-
ttio ,?)'er^0?ked by the bulk of the profession than this?certainly one of the
tj 1 destructive to life if neglected beyond a certain period. I am glad to notice
j. ~r. Stokes makes a similar remark. Some fault attaches indeed to the sys-
, 1 a*lc writers alluded to, for their mistaking the state of anaemia, with its quick
th 'Se' ^or irritative fever, by which they not only mislead themselves but also
t, eir readers, as to the nature of the patient's condition, and, consequently, as to
fav aPProl'riate means of cure. It has resulted from this, that a far too un-
tj ?Urable impression of the curability of chronic pleurisy with effusion, or em-
)?ma, as it is called after a certain time, has become prevalent. Dr. Law thinks
yre favourably of the possibility of cure. He with justice, however, excepts
he -ercuJar cases, and those in which the patient is not assisted; yet I think that
18 mistaken in supposing that a copious evacuation from some other organ
y ^?t occasionally prove critical and empty a chest. A case occurred to me
a ^7 V. ahsorption did not commence so soon as I expected : namely, within
nfe ' when the patient was attacked with liypercatharsis to the amount of 60
t\v ^ evacuations in two days. The chest, meanwhile, which was dull within
Inc^les ?f the left clavicle, and had the heart protruded to the right side of
sternum, had completely emptied itself, and the patient recovered*
^ r?ussais met with only one favourable case out of eighteen. Laennec's view
f ^ equally gloomy, and Dr. Townsend's is no less so; Dr. Thomas Davies
eis the same so strongly, that he hurries on the operation of paracentesis at a
*7 early period of the disease?a circumstance which is the main cause of the
j.nusual success of the operation in his hands. From this aggregate of un-
bearable opinions it results that, at the present time, there is a prevalent doubt
? ether the fluid of empyaema is ever absorbed. This fluid, it may be remarked
^ Passing, may be either sero-fibrinous and albuminous, or contain pus in any
j^gree up to its pure condition. This seems to be now a settled question, and
i ;llnk it ought to be so, as the fluid, in healthy subjects, kills not by its quality,
u by suffocating.
j cannot feel surprized at this want of success in the cure of empyaema, when
^n?tice the unsettled, vacillating, inadequate treatment recommended even by
?,S"e writers who think most favourably of the possibility of a cure.
jje . r* Law's treatment comes nearest to that which I have found effectual, but
irr'f8 t0? ^hnid in continuing the gentle use of mercury, from fear of its inducing
c ative fever and hectic. This supposed irritative fever, however, is, in most
nothing more than excitement of anaemia, (a fact of which he does not seem
as Je aware, as even in the convalescent period, he does not even name iron
a remedy,) and the hectic is a necessary consequence when the fluid is pus,
296 Extra-Limites. [Juty *
and this is diffused through the whole circulation by the process of absorpt10^
I have steadily continued the gentle external use of mercury through the ?
violent hectic, coming on twice a day in tremendous paroxysms; while 1?a ,
counteracted this by the free use of mineral acids; and by a diet, not only
strong broth at luncheon, but of animal food at dinner?the patient's tong11
being clean, and his appetite and digestion always good. . ?)
Dr. Townsend seems principally to follow Broussais (phlegmasie chronique'^
and Laennec, neither of whom make use of mercury, and the former would on /
venture on a blister as an experiment! He, likewise, falls into their great err
of mistaking anaemia for fever, and, therefore starves the patient at a mome
when there is a great demand for animal nutriment in any way in which it c
be borne. The treatment of Dr. Thomas Davies is that of calomel and opi? >
and counter-irritants in the first stage, but he thinks these inefficient in the stag,
of chronic effusion. He, therefore, as already stated, hurries on the operation
empysema. The writer on pleurisy in the Library of Practical Medicine (Vol-"
p. 124) seems to have but an indifferent opinion of the curability of chronj
pleurisy with effusion. After the third week or so, he thinks mercury of W*
benefit, and that it is even injurious when the hectic stage comes on; but ap'
proves of counter-irritants, and follows Dr. Stokes in his approbation of the
of the hydriodate of potass, to act both as an alterative and a diuretic; also 0
the iodide of iron. .,i
Dr. Stokes, whose writings on pleurisy I had not the pleasure of seeing " ^
long after I commenced my own observations, I find to be far the most success'
ful in his treatment of chronic pleurisy and empysema. In an excellent chapteI^
containing a considerable portion of original matter?some, perhaps, a little fa?"
ciful?he mentions that he cured twenty cases running by the use of a pint da]';
of cold solution of Lugol's iodine, and from a quarter to half an ounce of t?,
ointment rubbed into the side. He is, likewise, very favourable to the use 0
blisters.
I have myself been instrumental in curing five and thirty cases consecutive!)''
during the space of four years, but principally two years and a half, while I v'aS
assistant-physician to St. George's Hospital, no cases having been withdraw?'
or added from an anterior date, except three; the 1st was Mr. Garnett, who?1
I saw about 1833, who had also fatal ulceration of the bowels; the 2d, the Ke;v'
Mr. , whom I saw about 1833; the 3d, an out-patient of St. George'?'
whom I found to have tubercular disorganization of the lungs, and whom *'
therefore, transferred to Dr. M'Leod, as an in-patient of St. George's. Pa1*'
centesis was practised; the tubercles were found; and he died from inexpansio?
of the lung; which was bound down to the spine. The remainder of the cases
all dated within three months, as well as I could make out by most carefully,
catechizing the patient respecting the first feeling of pleuritic pain or ailment oi
any kind. The pain was frequently forgotten, until the patient was perhaps
asked whether he had not had a little lumbago, pain in the back, &c. Nor lS
this surprising; for copious effusion very soon relieves pleuritic pain. A very
great proportion dated within two months, and from that time "down to three
weeks or a month. I seldom saw them earlier than a month, as they were either
neglected and misunderstood cases amongst the out-patients of St. George's, ?r
private patients whom I was called to see in consultation at a late period of the
disease; the complaint of the latter having, with few exceptions, been also over-
looked.
The following is a list of the previous duration of the disease in all my private
cases, amounting to seventeen; but I lament to say that I cannot at present
the dates of those, eighteen in number, who were out-patients of St. George's>
and the notes of whose cases I drafted out of 15,000 notes of cases, which I sa^
at St. George's, (for I took notes of almost all.) The .notes of these eightee?
cases having been separated from the others, I have unfortunately mislaid thei?-
^41] Treatment of Chronic Pleurisy. 297
for t^SS' t^iere^ore' I recover them, I must trust to the confidence of the public
tkQ .e ^ccuracy of the facts. They were all demonstrated, as they occurred, to
'be students of St. George's.
oachman of Sir Clifford Constable's, ill a fortnight, but previous bronchitis,
p Caldow, ill from two to three weeks.
v, er* Watts, ill eighteen days.
trr- Smith, ill three weeks.
r. / apson, ill a month.
v^ripk Millerick, ill a month.
ti ?r" Eade, ill five weeks.
V.r> Garnett, ill six weeks
enry Wade, ill two months.
AH Wvn^nS' two months.
uersgate Street Student, under Mr. , for supposed phthisis?had sup-
- posed lumbago nine weeks before.
^ Miller, disease of ten weeks standing for two months.
AT 6 uV" .^r' ?arter' two months and a half, but previous pneumonia,
p.f- Hamilton, ill three months.
liza Gray, ill three months.
It!"- ^1 organ, stitch in back three months before. Ill ever since.
he Rev. Mr. ? ill upwards of three months.
s 1 have not leisure to continue this paper at present, I subjoin the following
rj??randa of how I shall proceed if time permit.*
se Vr Pr*vate cases, being in great detail, and in general features greatly re-
Pirt- ? each otber, it would be useless to give the whole in full. Therefore
Mr vUt a *"ew which siye at length, as general types?for instance, Miss Miller,
a r'^Iorgan, and Sir Clifford Constable's coachman?the remainder insert in
re a y^eviated form, together with such of the out-patients of St. George's as I can
t0 memory> though I have lost the notes of their cases.?Shew that I used
eff r?Ury in all degrees of intensity, so as to ascertain what quantity was the most
in ^ut' at the same time, least injurious.?Shew that I always used opium,
for ProI?ortion, with the mercury, and that I used the milder and the external
the'118 W^en the others could not be borne?thus taking especial care to protect
*uc?us membranes.?Add that I found prompt and free salivation by calo-
atl(| and opium, and the use of one or two drachms of ointment on each groin
oth axi^a night and morning for forty-eight hours, (in conjunction -with the
efF 6r reme(lies presently to be specified), produce the most rapid and satisfactory
c'ts of absorption, in cases where the dyspnoea and faintness seemed to me
st urgent and dangerous. It was quite common, and, in fact, occurred in the
do J?rity of cases, that the fluid descended one-third, and still oftener one half,
thWn t^e chest, within the space of forty-eight to sixty hours, carrying with it
? extreme dyspnoea and faintness, to the great relief of the patient.
SetJ.ay that blisters were used from the first, and that the following became my
C] . Plan of managing them. One blister 6 inches long, and 3? broad, ex-
theS1VC mar&in> was placed longitudinally over, and a little to the outside of
aH(l*lI>fles t^e "I58' leaving space for another of similar size between the first
? the spine. Great care was taken not to remove the cuticle, (one means of
far v1 was to cover the surface of the blister with silver paper,) as this forms by
the quickest healing plaster; but after about forty-eight hours, during which
be inning was absorbed by dry napkins, carefully prevented from adhering, it
requisite to cover the whole with the mildest soap-plaster, spread on soft
c?' to prevent the cuticle from being accidentally abraded. In this way all
tec] ^ be observed that, from this point, the form of private notes is adop-
298 Extra-Limitks. [July
irritation promptly subsided, that is, in the course of from two to three day ^
and the patient was ready for the second blister, which was placed between 1
first and the spine. It was similarly treated; and, at an equal interval, a tW
was placed in front of the original one; that is, rather forward in the axiJ '
When pain indicated the possibility of a pleuritic stitch in any part of the si >
it is needless to say that the first blister was placed over that.?Say that diureti
are conjoined: viz. Squill; Sp. ./Eth. Nit.; juniper; iodide of potassium, an '
when there is no irritation of the mucous membrane, the various other preI'a_
rations of potass. Digitalis, by creating faintness, is apt to confuse the syini'
toms; I do not therefore, use it till later. Where all these remedies had falle ^
for two or three days, and dyspnoea continued as urgent as ever, I have occa
sionally used a powerful hydragogue, as half a grain to a grain of elateriu111'
combined with calomel and capsicum to prevent nausea; or the Pulv. JajaP'
Comp. 3j.; so as to produce ten or twelve copious watery evacuations per da)>
stimulants being at hand in case of any sinking tendency. The effect of t'11
has on several occasions been perfectly satisfactory, absorption in the cne?
having now made rapid progress. I derived this idea from a case already a *,
luded to, in which a patient had an accidental hypercatharsis to the amount0
sixty stools in two days, which emptied the chest in the same space of tunej
The patient is better in bed, both because it favours gentle transpiration an
obviates faintness. j
Remind that, hitherto, I have been treating a case in which the dyspnoea seeing
imminently dangerous, and the most vigorous use of remedies, consequent;
indispensable; but now explain that inconvenience sometimes resulted fr01
hypersalivation; for, notwithstanding an immediate suspension of the mercu y
either on the first appearance of tenderness of the gums, or of amelioration
the symptoms?especially the dyspnoea and obvious commencement of absorp^
tion, untoward salivation would occasionally occur and greatly retard the conV^n
lescence.?Explain that, on several times observing this and having reason
believe that the patient could bear the dyspnoea with safety for some hours long^1"'
provided he were prevented from rising, which creates faintness, (case of ^ '
Smith, barrister,) I used more moderate quantities of mercury, being content t
affect the gums within three or four days. In this way, the action of the remedy
was easily controlled, either by omitting the mercury for two or three days ifll
action threatened to be considerable, or by merely diminishing it according t
the evidence of the mouth and of the symptoms. I found, however, that it d><
not answer to suspend it altogether, but that a continuation of it daily in a xo?,
form, as a blue-pill night and morning, or at night only, for the purpose 0
maintaining the first impression for a period of two or three weeks, or, in sli?r '
until all the disagreeable symptoms had disappeared, was attended with far bette^
success.?Explain, further, that the great acceleration of pulse, which rises cofl}*
monly to 120 or 130, and in young persons even to 150 or 160, and which 1
attended with what the patient calls " internal fever," thirst, craving for co\
drinks, and dryness and heat of skin, is not necessarily a result of fever, but i
is necessarily a result of anaemia, occasioned by the deficiency of oxygenati?
from the total incapacity of one lung at least. Here was the error made 'y
Broussais who supposed this to be fever and put his patient on the lowest die*
On the contrary, acting on the opposite principle, I always supply my patien ^
with at least one or two pints of concentrated beef-tea, or plain ox-tail soUp^
and, if the state of the tongue and the alimentary canal fully authorizes it, 11"-'1''
mit them tender old mutton or beef for dinner. On this treatment, the pu's
and "internal fever" rapidly fall in proportion as the anaemia disappears. .
Next proceed to those cases in which hectic is established, resulting, for t'1
most part, I should imagine, from the fluids being of a puriform character J
after a month or six weeks, and sometimes much earlier if the inflammation ha^ ^
been very intense, it assumes this character.?Allude to the opinion pretty P,e
Treatment of Chronic Pleurisy. 299
m.ercury is injurious in such cases, and say that I have not found-it
been ( , *ts use was indispensable : for I have noticed that where it has
si0n j mit;ted, contrary to my wishes and instructions, a recurrence of the effu-
otherUlS ,.en P^ice, notwithstanding the use of mineral acids and the various
tnerCure|ne. es u?ua% considered available against hectic; whereas, on resuming
to rest^ Wlt^ ?Pium an(l giving the mineral acids for hectic, I have been enabled
t? ^ ore ,matters to their former condition, though not without an extra shake
Const P,at,lent- One of the best instances of this was presented by Sir Clifford
three , ,'s coacbman.?Dwell on this case; say that, after acute pleurisy of
Of^er^i I saw him, and the chest was emptied in a week, but the mercury
he s be continued. This, on my taking my leave, was omitted; and, as
ancj of'?e weak, (viz. merely from anaemia,) he was ordered ammonia, brandy,
reft]] i stimulants. In ten days, when I was again called in, his chest had
and ao-,,an^ now a most violent hectic paroxysm at eleven o'clock, A. m.
extreif- at M- Each of these thoroughly drenched him, and during the
18aw e beat the pulse would rise to 150, being also barely perceptible. In this,
endea n? . n? but a large quantity of pus in the circulation, which nature was
y ?Uring to throw off in her usual manner. I believed, in short, that the
blisteln ? cbest was wholly purulent. I, therefore, continued the mercury and
he 1) 18 ln m?deration and made free use of the mineral acids, which, fortunately,
^hih'1"6 'Jerfect!y well. During the brief intervals of the hectic paroxysms he
Petit 'f ^lat marked relief which we habitually see, and had always a keen ap-
?r r 6 meals. He, accordingly, took as much mutton-chop, beef-steak,
che^aSt or foiled mutton, as he was inclined to take. Under this treatment the
slowl ,Was aSain emptied within the space of ten days; the hectic symptoms
Cot) y Save way during a period of a month or six weeks, and I dismissed him
Was escent to go to the country. I may add, however, that when the hectic
eo.onearly &?ne, sulphate of iron was added to his sulphuric acid in order to
jy ei"ate with the animal food in removing all remains of anaemia.
liighlM likewise on the case of Mr. Morgan, ret. about 20, who was not only
ternai ^lect*c> but had also slight gastro-enteritis. I continued the gentle ex-
gastro ^ ;incl. mutton-broth, in such quantities
rniner "1ente.r^is having been thus pretty well subdued, he became tolerant of the
reri()e'! acids and sulphate of iron, well guarded with laudanum. This youth
the fl ^le progress of absorption more rapid than in the preceding case, for
s? ?r u disappeared within ten days, with entire relief to his dyspnoea; and
a res^orati?n resulted from the new supj)ly of oxygen and removal of
to r>^nlEi' ^lat aweek afterwards he came in a cab from the City to the West-end
jp1 upon me.
t? Scuhators should be careful not to throw away their chances by neglecting
eXa ? "'e stethoscope. In one instance, an accomplished physician, having
i^dur - *?P tbe lung and found it dull, with the other usual signs of
case fatlon' without following up his examination down the whole side, took the
friend0}! P. bisis, and ordered the patient to the southern coast. A common
at 0n "av'ing mimicked to me the mode of breathing of the patient, I declared
of pw'- f. r bis imitations were most graphic,) that such wa3 not the dyspnoea
in r()i Usis!' an(i as I knew him not to be a phthisical subject, and to have been
patient ^h two months before, I entreated of our friend to go down to the
sta^ t -ln /^le country the same afternoon, and not to let him stir (for he was to
self; ,ys after) without a consultation of physicians. I declined going my-
8?> hfS v*nS suggested the measure. Answer was returned that if I would not
ine> _AVould see no one else, as he had originally intended to have consulted
?i(je savv him and found?what I was before sure of: namely, that the whole
full of fluid, indicated by the usual symptoms, including anaemia and
300 Extra-Limites. [July
the physical signs. I flattered him that we should empty the chest within a
week or ten days, and that he would be convalescent, Deo volente, in a .
So it happened, though mercury could only be borne externally, and that vn '
great reluctance on the part of the patient. His convalescence was somewh^
protracted in consequence of the irritable state of the mucous membranes, rei1'
dering them incapable of bearing the animal diet, and ferruginous preparation
suitable for the removal of the anaemia. He had flying pains principally
the region of the heart, but these ceased under the use of plasters, especially bell3'
donna; and he has enjoyed perfect health for the past year. .
In the great majority of cases an attrition murmur, (always most percept"*
along the line of the margin of the lungs from the heart, curling backwards
the bottom of the lower lobe; in other words, below the axilla,) was found
appear as the fluid disappeared by absorption. I have noticed that the longe
this attrition murmur lasts, the better; as the adhesions are more apt to be of ?
loose and elongated character, which I infer from the patient's recovering coin*
plete resonance on percussion, and complete restoration of respiratory murfli?
sooner than in other cases where the attrition murmur lasts but a few days,in
consequence, probably, of the adhesions becoming universal and close. "When*
ever the latter is the case, the patient may lay his account to being more or jeS
delicate for a year and a half or so, because the lung requires fully this tim?
slowly to stretch the adhesions, to re-acquire her natural respiratory murmur>
or, if this should never occur, for the patient to gain a compensation by hyper"
trophy of the opposite lung, which, meanwhile, has constantly been doing vica'
rious duty; namely, breathing in an exaggerated or puerile manner. The8
exquisite arrangements of Providence cannot be sufficiently admired. The ??r
we look into them, the more complete we find them. ,
The lung sometimes remains permanently condensed from the thickness an
utter inexpansibility of the side : and dilatation of the bronchi may result fr0111
this cause, of which I have met with and detected four or more instances. C?n'
densation of this kind is less frequently attended with falling in of the side tha
in cases of pleurisy; for the opposite lung slowly becomes hypertrophous a11
fills up the vacant space, advancing, however, into the opposite side of the ches{>
and carrying the heart with it in either direction. Thus, in Peter Parker, an
out-patient of St. George's, the heart is protruded almost into the right axilj3'
and the aorta pulsates an inch to the right of the sternum. Lung condensed Pj
adhesion, is rarely healthy. There is almost invariably a slow, wearing, chr
bronchitis, which harasses and reduces the patient, and generally curtails his e**
istence. Parker had had his cough for ten years, and he was an emaciated an
decrepid subject.
Here introduce a number of detached observations, more or less original,
various subjects. Thus, for eight or ten years, I have been in the habit of asking
the question of all respectable patients of robust constitutions, who had bee11
attacked with pleurisy, peripneumony, acute rheumatism, whether they were
the habit of wearing flannel, or not; to which they generally answered in 1''
negative?the common reason assigned being, that they were so much expose"'
that they could not venture to pamper themselves. I recently put the sa?<j
question to a London physician, and he gave the same answer with a smile, an
" my dear friend, it is impossible, &c." He was attacked with rigors the sa^1?
night, and had a severe rheumatic fever. I do not quote the poorer classes,
they, almost universally, are deterred from wearing flannel by the expense,
it is notorious that they are subject to acute inflammations of all kinds, in
much greater proportion than the higher classes. Flannel is also highly bene
ficial to chronic affections of the mucous membrane of the lungs.
Pleurisy is, after rheumatic fever, one of the most frequent causes of peri car-
ditis?not endocarditis, at first?the inflammation being propagated to the perl
184]]
Treatment of Chronic Pleurisy. 801
f'T ^ .c?ntiguity of tissue : if endocarditis supervene, it is by propagation
jy "le peri- to the endo-cardium.
acti laP aSmatic pleurisy may occasion agonizing pain by interfering with the
siorj0?] S? ?reat a muscle as the diaphragm. It is in such cases that we occa-
Path ? see the patient put on what is called the sardonic grin, a species of sym-
etic spasm dependent on the excito-motory system and nerves.
s, n some convenient part of the paper, give a brief and compact, but very clear
j-n ?Psis of the signs of a chest full of fluid. For I repeat that, well as these are
Pra V"11- systematic writers, they are singularly forgotten and overlooked by
0c ctl.tloners. They ought, therefore, to be pushed prominently forward on every
thafS1?n'- ^e^er' likewise, to a synopsis of the signs of anaemia in the essay on
fro S,ukject; by which the practitioner will readily distinguish this condition
\v* fever, for which it has been mistaken by Broussais and others.
Iin -n^ UP })y a general statement, that, if Dr. Stokes has cured twenty cases
ari(jtllnS by Mons. Lugol's solution and ointment of iodine, together with blisters
other means; and I have cured thirty-three consecutive cases by other
p ns 5 fifty-three cases cured successively, without selection, afford a strong
re HmPtion that all really curable cases are curable without paracentesis. It
: . ains to hp nrnvprl hv pviipripnrp whether iodine or mercury be the less in-
jur)iaiI1S to Prove<i by experience
har?^S t0 ^ie constitution. I have myself the most favourable opinion of the
litti vSSIless ?f the iodide of potassium when protected by starch?that is, a
d0 6 ead with each dose; for I made the experiment of giving eight-grain
ta; es against three-grain doses, in two hundred cases, for the purpose of ascer-
a ?lng which dose was the most efficacious. The larger, both being given thrice
jay> succeeded incomparably the better, and I now rarely give the smaller,
a y, ? met with seven or eight cases of circumscribed pleurisy, and whenever
San l)leurisy becomes very protracted, I am not sorry to see a purulo-
chi ^Ulneous discharge take place periodically, as it generally does, into the bron-
Pat tu^es ' f?r> in this case? a slow process of healing generally occurs, and the
jj lent> in a fair proportion of cases, recovers. I have recently discharged one,
prenry ^ ade, who appeared to me to have had a chronic pleurisy engrafted on a
de\M?US c'.rcumscribed or sacculated one. The history of the chronic one is
that ?Ped *n my journal in the utmost detail; but the patient also informed me
Mi i?me mont^s before he had been under the care of a physician in Norfolk,
pus ? treate(i him f?r six months previously for a discharge of half a pint of
Aft rni^e^ with blood, expectorated once a day from the bronchial passages.
Vei>r ? 8 t*me sent ^im to London to consult me, and I found him with a
v, y Clrcumscribed empyaema that he might have had, obscured by general em-
at tima" '^1*s living been removed, the original circumscribed empyaema pointed
W; r c^est5 and discharged by two or three apertures. When the discharge
t * *ree by the apertures it was correspondingly diminished by the bronchial
es- Both slowly decreased. The circumscribed empyaema seemed to des-
as Ver>' low in the splenic region, and after nine months of hospital attendance
Reiian ^"Patient he was dismissed without discharge, with slight cough and in
j^a era^ good health, his weight being at least 12 stone, though a moderate-sized
J discharged a patient from the Mary-le-bone Infirmary, cured six times of
At tVrriscrih>ed empyaema above the left mamma, and opening into the bronchi.
j le erjd of six months he was completely well.
}Je n another, in the Mary-le-bone Infirmary, the whole length of a probe could
s;(jPassed directly into the chest. He recovered, but with much collapse of one
third in the Mary-le-bone Infirmary, a boy of 18, had effusion for six
tui ls- My colleagues, in consultation, had given their opinion that^ he was
The operation of paracentesis, therefore, was negatived. The op-
e *ung was soon after attacked with peripneumony and he died, when the
302 Extra-Limites, [July
exemption from tubercles was proved. I did not tlien understand an e^c'^L
treatment for fluid in the chest; and was therefore an advocate for the eariy ?Pj
ration on the principle of the late Dr. Thomas Davies of Broad Street. 1
much regret, therefore, respecting this case, that the operation had not been I
formed. One feature was remarkable. Andral, Broussais, and others, rec
mend that patients in chronic pleuritis should be kept on light diet. This y?" q
ate twelve ounces of dressed meat at dinner; eight ounces at breakfast with
eggs; tea and milk ad libitum; sixteen ounces of bread daily. . ?
Numerous similar cases shew that nature's mode of performing the opera
is incomparably more safe than paracentesis.
1841]
On the Poison of Fever. 33
attack ?^ec';'on assumes that typhus confcrs no immunity from subsequent
ex .s an^ that the exanthemata do confer an immunity. The answer is, that
to warrants our belief in a considerable power of destroying the liability
ex , seclUent attacks in typhus, and that, though there can no doubt of the
It ernata possessing this power, exceptions to it are frequent in all of them,
tecti mus* he admitted that in this country there is a general belief in the pro-
a n? P?wer of a seasoning or initiatory fever, and though we rarely meet with
hav Tal man W^? ^as n0t tyP^lus* we certainly meet with few indeed who
pre more than once. The nature of the subject does not admit of very
, Se Pr?of. We can only obtain the general results of experience. Dr. Barker*
es as the results of his, " that he has for some time entertained the opinion
sec S^ere.rs from fever attended with this eruption, if they are not altogether
hav k k"T ^ fr?m a second attack, are not at least so liable to it as those who
en e. a(l a fever of the ordinary kind ; and, though he frequently made the
sJUlry> he never found a patient in whom this symptom was distinct who had
res e.'"e^ from the same fever on any former occasion." Dr. Perryf states as the
js , , an extensive series of observations his opinion, " that typhus generally
fre 60 once a 'ife-time, and that a second attack does not occur more
^quently than of small-pox, and less frequently than of measles or scarlatina."
sta't i V' on states that of 609 patients in the Glasgow fever hospital only 74
^ 6 '?hat they had ever had fever previously. He justly observes that when
thi 'nt? account the various diseases which are confounded with typhus,
small number can be easily accounted for.
in Pr?tective power of the exanthemata has been much overrated. Three
ances of second attacks of small-pox came to my knowledge in this county
I r- recently. In two of them the patients had suffered the disease from inocu-
a IOn very few years before. In one in which the inoculated disease was severe
\r^l0,st confluent eruption accompanied the second attack seven years after,
ha ?[l ^stance has been related to me of a lady living in this country who
. s had the disease three times. Dr. Roupell refers to the case of one who had
II seven times.
f;v n?tances of second attacks of measles are given by Dr. Bailie, who attended
JLchildren in May, and again in the following November, by Dr. Webster, J and
in W^? s*-ates that he met with three instances of second attacks of measles
, interval between the publication of the first and second editions of his
be [ ^'le remarkable case of a second attack by Dr. Graves ? should, perhaps,
aft errnec' relapse into measles. The second eruption appeared twenty-one days
r e.r the commencement of the first illness, in which the eruption had been
P'?us and severe.
wses of second attack of scarlatina are stated by Roupell to be not at all
common. Several have fallen under my own observation.
t * ? The liability to relapse in cases of typhus has been urged as an objection
the classification by Harty and others. It might be replied that cases of
. easles, such as that of Dr. Graves just referred to, and cases of reversio, as it
termed by Rayer, after scarlatina, would tend to shew that the exanthemata
G n?t exempt from relapse. But the true answer is that typhus is peculiarly
j, 'npt from relapse. Two kinds of cases are erroneously considered such : 1st.
apses from typhus into fever symptomatic of a visceral irritation?generally
stro enterite.?" I am persuaded, says Cheyne, || that obstinate and fatal
. ^ublin Medical Transactions. Vol. 2.
7 ^ubhn Med. Journal, Vol. 10, and Edin. Med. and Surg. Journal, Jan. 1830.
t i^dico-Chirurgical Transactions. 2nd Series. Vol. 4.
Ij i^ublin Medical Journal, Nov. 1S40.
" Dublin Hospital Reports. Vol. 1.
34 Extra-Li mites. [July 1
relapses after typhoid fevers are often attributable to inflammation and, perhaps*
ulceration of the villous coat of the intestines." And Broussais asserts tha
"when the frequency of pulse in 'convalescence' does not diminish, and tn
strength does not increase, it may be suspected that a form of latent inflamma-
tion exists. It may be discovered by permitting an excess which general
changes this frequency into a real fever, and developes the pain of the irritate
part but, 2ndly, cases of fevers not typhoid will under exposure to infection
relapse into typhus. Dr. Davidson gives a tabular view of the relapses in
Glasgow Fever Hospital among 686 cases, in which no case of relapse from
typhus into typhus occurred, but two of febricula and one of intestinal fever
into typhus.
In 500 cases of fever admitted into the Navan Hospital in 1840, two case
only of relapse into typhus occurred, both were cases of febricula, which alte
a few days were sent to the convalescent ward, where they relapsed into macu-
lated typhus, one in four days and the other in fourteen days after their rem?va
thither.
VI. Lastly, the following extraordinary objection is put forward by Dr*
West:?f
"The type of the fever itself varies, being sometimes intermittent, sometime'
continued, changing from the one to the other form, and being occasionally
converted into other diseases."
In other words there is no such disease as typhus!
To this the supporter of the speciality of typhus replies, that the disease's
here, and in numerous quotations throughout the paper, confounded with other
fevers ; typhoid it may be in their nature, or becoming so in their progress, no
arising from an animal infectious poison, but from a variety of sources, whic
contain a variety of poisons, the identity of any one of which with the typh'lS
poison is a matter in dispute, and to be argued upon the conclusion ol ?ur
investigation into these sources in the following chapter.
Meantime the pertinent remarks of Dr. Copland, upon this subject, are n?
unworthy the notice of those who rely for the means of drawing accurate dis-
tinctions upon such sources as Dr. West has explored:?" True or contagi?llS
typhus has been confounded with synochoid and nervous fevers on the one
hand, and with putrid or malignant fevers on the other. It has been already
stated that putridity or malignancy, not only may characterize a particular form
of fever or certain epidemics even at an early period of their course, but, also*
owing to various contingencies, may take place in advanced stages of any other
fever. As the circumstances favouring the generation and spread of typhus are
often such as also tend to develope those changes which have been usually name"
putrid or malignant, and as these changes are frequently observed in the latt^
stages of typhus?the symptoms distinguishing this fever becoming associate"
with, or followed by, those indicating the putro-adynamic state?so has it been
often confounded with other fevers in which this state has predominated more
or less. If we refer to the numerous histories of epidemic typhus, recorded bv
writers from the close of the 15th century up to the present time, we shall find
that although many of these, owing to the concurrence of circumstances, deye'
loping a putrid or malignant disease, were instances of fever either identical vvito
or very closely resembling that which I have described as such in the preceding
section, yet many others, or even the majority, were true typhus, in which the
putro-adynamic state was either early or predominantly developed. The exan-
thematous eruption, characteristic of typhus, being succeeded or accompanied by
the petechise, indicating the approach of the septic condition, and being either
* Chronic Phlegmasia, Vol. 2, p. 53.
f On Exanthematous Typhus.
1841]
On the Poison of Fever. 35
esDe^n0 *"?r ^em or f?r an eruption of miliaria. Owing to this circumstance,
foiinrij typhus, low, nervous, and putrid fevers, have been very generally con-
"nied together."
rabl1C reac*er foregoing, and many other passages in Dr. Copland's admi-
Pell'0 ai tlc'e on typhus, must be startled with the following passage in Dr. Rou-
pe Jj recent work on the disease, when he finds that Dr. Copland is not like
8il es and others, who have described exanthematous typhus, passed over in
ferre^t ^Ut 'S actua^y mentioned by name as belonging to the authors re-
fev a^ove description typhus is considered to belong to the continued
HotrS' ^ 'S '00'ced upon by the most recent authors, in this and other countries,
ou &n '"dividual disorder, but as one into which others may be and fre-
jj y are converted !" (page 5.)
for ^?r t^le Prc,ent we leave the suoject, since that portion of the argument
diff 16 c'ass'fication of typhus with the exanthemata, which is derived from the
sid6^1-068 between it and other continued fevers, will properly come under con-
ation when discussing the identity of typhus and typhoid fever.
Q
chapter II.?Of a Fe veu Poison, generated during the Decomposi-
, tion of Dead Organic Matters.
g le difficulties attending an examination into this part of the enquiry into the
th r?eS ^ever are very great, and are confessed by all who are familiar with
b f ^Aicting statements advanced on the subject. Our difficulties are increased
nL indeterminate character of much of the evidence offered in proof of the
st v, or'?'n fever, some of which not only claims to prove the power of
tlii s?urces to cause continued, but infectious fever?by the fact that much of
jn s.evidence is moreover inadequate, as it proves only the occurrence of fever
sltuations and among persons which might be considered equally obnoxious
jatiContagi?n as to miasm, and by the silence, or mysterious, or contradictory
s ^u.aSe of those to whom we might look for assistance and direction in a
beU?y of the mass of conflicting testimony, from which our conclusions are
; ,^Us> Dr. Christison says, "the great questions involved in the investigations
Rin? ? causes continued fever are three in number :?Does the disease ori-
ate in infection ? Does it originate in other causes ? Granting that it does
jj. S'nate in other causes, may such fevers propagate themselves by infection ?
fir)AV'" seen that they cannot be all answered by any means with equal con-
<ence ;" and, accordingly, while he is full and illustrative on the subject of
"tagion, he treats of olher causes in a most cursory and unsatisfactory man-
er> and while he admits that " the general conclusion from the whole facts
emst? be that a disease, undistinguishable from true infectious fever, may
betimes arise without infection," adds, " that on descending from the general
be pSt'on to the more special one?what the other cause or causes of fever may
?ty-'r-the difficulties are greatly increased, indeed they become insurmountable,
ject ,?Ut such limited and vague facts as are at present possessed on the sub-
fi, ^ and " it appears a needless waste of time and labour to attempt anything
Ur*her on this head."
j ?r are we more enlightened by Dr. Davidson, who, while he states that he
st Prepared to assert that febrile affections may not, under peculiar circum-
exH?eS ^w^at he does not inform us), arise from paludal sources, effectually
u udes them from consideration by putting forward the following conclusions
head of "Alleged sources of continued fevers, not typhoid."
sn ?0In a consideration of the whole evidence that might be adduced re-
nting this point, it may be drawn as a conclusion, that although putrid
C 2
36
Extra-Li.mitks. [Jaty ^
matters when injected into the veins of animals cause death under syropto?
similar to those of typhus fever, yet that the effluvia arising from similar rna *
ters do not, under ordinary circumstances, produce any deleterious effects
man." Again?"Before concluding this part of the essay we shall notice a
hypothesis, which has lately been somewhat confidently brought forward
account for the prevalence of typhus in some large cities, viz. : that a peculi ^
malaria is generated by the animal and vegetable filth, which accumulates alon<3
the sides of rivers-running through large towns, and that the inhabitants w
live in their immediate vicinity become thereby subject to fever. We are qul
aware that very disagreeable and sometimes fajtid effluvia occasionally arise fro
such situations, particularly during hot weather, but that it is capable of causing
continued fever has not even been rendered probable by any satisfactory evl"
dence."
We have it stated upon high authority that gaseous contagions contain organ'
matter in a state of decomposition or progressive change. We have it also an-
nounced that from certain decomposing animal and vegetable substances, organ1^
matter in a state of "progress to decay" is evolved, which, when collected an
retained in a manner similar to the former, completes the stage of decomp?sl*
tion, or, in other words, "putrefies." ,
By evidence of the most unexceptionable kind the former of these is prove
to be capable of communicating the state of change, of which it is the subject,
the healthy human organism. t
We have to enquire whether the analogy of action of these bodies is as perfe
as the analogy of condition appears to be, and, whether, " when the process
respiration is modified by contact with a matter in the progress to decay, w ,
this matter communicates the decomposition, of which it is the subject, to t11
blood?disease is produced." ,
We shall first state the analogy of condition of the tangible poison, evolve
from decomposing organic substances, in the words of Dr. Southwood Smith""
not only because it is clearly stated by him (so far as relates to its tangible e*lS'
tence), but, also, because this passage has furnished the text for some of
objections which we shall have to consider* :?" It is known to every one that tn
putrefaction of vegetable and animal matter produces a poison, which is capa?'
of exerting an injurious action on the human body. But the extent to
this poison is generated, the conditions favourable to its production, and tlj
range of its noxious agency, are not sufficiently understood and appreciated. *
is a matter of experience, that during the decomposition of dead organic sub-
stances?whether vegetable or animal?aided by heat and moisture, and otne
peculiarities of climate, a poison is generated, which, when in a state of ?l8
concentration, is capable of producing instantaneous death by a single inspira*
tion of air in which it is diffused. Experience also shews that this poison ev^11
when it is largely diluted by an admixture with atmospheric air, and wheD'
consequently, it is unable to prove thus suddenly fatal, is still the fruitful sonrf
of sickness and mortality?partly in proportion to its intensity, and partly 1
proportion to the length of time, and the constancy with which the body remain
exposed to it, &c.
" But this poison was too subtle to be reduced to a tangible form. Even ''
existence was ascertainable only by its mortal influence on the human body ;
although the induction commonl'y made as to its origin, namely, that it is
product of putrefying vegetable and animal matter, appeared inevitable, seeing
that its virulence is always in proportion to the quantity of vegetable and anin?f
matters present, and to the perfect combination of the circumstances favourab
to their decomposition, still the opinion could only be regarded as an inference'
Poor-law Commissioners' Fourth Report, page 130.
1841]
On the Poison of Fever. 37
?ut modern sciencc has recently succeeded in making a most important step in
e elucidation of this subject. It has now been demonstrated by direct experi-
ment, that in certain situations, in which the air is loaded with poisonous exha-
ations, the poisonous matter consists of vegetable and animal substances in a
state of putrescency. If a quantity of air in which such exhalations are
Present be collected, the vapour may be condensed by cold and other agents, a
j^siduum is obtained, which, on examination, is found to be composed of vege-
able or animal matter in a high state of putrefaction. This matter constitutes
a deadly poison. A minute quantity of this poison applied to an animal, pie-
v'?usly in sound health, destroys life with the most intense symptoms of malig-
nant fever. If, for example, 10 or 12 drops of a fluid, containing this highly
Putrid matter, be injected into the jugular vein of a dog, the animal is seized
^ith acute fever, the action of the heart is inordinately excited, the respiration
ecomes accelerated, the heat increased, the prostration of strength extreme, t e
jouseular power so exhausted that the animal lies on the ground wholly unable
to ot;_ _ . - - -- -
make the slightest effort, and after a short time it is actually seized
Which ? k'dck vomit, identical in the nature of the matter evacuated with that
inte -1S ^rown UP by a person labouring under yellow fever. By varying the
of a|S'^ and the ^ose the poison thus obtained it is possible to produce fever
I an^* ^'Pe' en(lowed with almost any degree of mortal power."
tion sen^ence we recognise the echo of Majendie's questionable asser-
^ s the preceding statements are confirmed by the account of experiments
fro n "^e niauvais air," given by Devergie :*?"The gas, which is disengaged
'in? Pu,tretyinS animal matters, extracts with it a particular odour, infectious
ecte,' characterized by the general term putrid odour. We attribute this
r r to miasma, that is to say to a cause void of meaning, because we are igno-
?f the nature of the object which it represents.
untz has endeavoured to enlighten the phenomenon by the following experi-
a m": P'acecl a be" g'ass over a portion of a putrefying dead body, in such
tem anner as to permit the air to penetrate, he submitted the apparatus to a
fici ^e!^ure ?f 26? Cent, (equivalent to about 78? Far.) and, after a period suf-
?f th ' Pro'on?ed, he suddenly cooled the bell glass ; immediately the product
asm 6 vaPour assembled itself into drops, which evolved a strong odour of mi-
?VVasa' he treated these drops with chlorine, when the odour disappeared. He
jjj , lus led to suppose that the gas in escaping from the putrefying animal
0f e.r carried with it the vapour of water combined with a certain quantity
animal matter, very minutely divided, and this constitutes what has been
Th miasma-
? "is is not the only experiment calculated to lead to this opinion?others have
Qf-en made with respect to vegetable matters. Moscati entertained the first idea
. c?ndensing the water dissolved in the atmosphere, for the purpose of detect-
S the principle which occasioned "le mauvais air." He suspended at some
^ stance from the soil mattrasses full of ice ; the water which became deposited
g' ?n their surfaces condensed itself readily, when limpid it presented many small
a f es vvhich possessed all the essential properties of animalized matter. After
^ w days they putrefied completely. In the course of the year 1812, M.
the Unc*erto?k> 'n the marshes of Languedoc, a series of essays directed to
bv fV016 enc'' condensed dew on glasses, and the water which he obtained
y nis means presented all the phenomena obtained by Moscati.
0j. n 1819 M. Boussingault observed that sulphuric acid placed in the proximity
Pidf 'n which he had caused animal matter to putrefy, blackened very ra-
y- He repeated this experiment in many infectious places, and found con-
rid* ^Iedicine Legale. Tom. 1, p. 100. I am indebted to my friend Dr. Aid-
Sc lor referring me to this account of experiments on the subject.
38 Extra-Limites. [Juty
stantly that the coloration of the acid was more prompt according as the air ^ a
more infectious," &c.
The inference naturally deduced from such experiments as the foregoing, take
in conjunction with the fact of the occurrence of fever in situations where the5
putrid exhalations have been found to exist?namely, that they contain a Jel*
poison?'has been met by numerous objections. The principal seem to be t
following:?1. That a mephitic poison is confounded with the fever poison*
2. It is denied that these sources ever generate fever, because the number 0
cases does not bear a sufficient proportion to the number of instances of exp?
sure, and because they generate several diseases differing in their nature fr?,
fever, and it would amount to a confounding of fever with these,* if we attri-
buted its origin to the same poison. 3. It has been objected to the evidence
the frequent occurrence of fever from this source?that it is furnished from to
experience of persons who deny the infectiousness of fever, and is, therefore
suspicious ; f that in the recorded cases malaria and contagion have been con-
founded ; that it amounts only to a proof of the frequent coincidence of fever
and the effluvia from filth, and does not prove that the former stands to the latter
in the relation of an effect to a cause; that, granting continued fever is ever tin15
produced, it is not contagious or typhus fever, &c.
I. It has been saidj?" If the statements of Dr. Smith were put into the
simple form of the only proposition which they really contain, they woul
amount merely to this?that exhalations from certain putrescent matters have
the power of producing both asphyxia and continued or typhus fever ; to
former of which is a result familiar to all, and the latter, a mere assertion, de-
riving a little hue of probability from its juxta position to a known truth. There
is a wide difference between the asphyxia, which is caused by mephitic gases,
and typhus fever,?a difference which can never be explained, as Dr. SiDitn
attempts to do, by a reference to the diversity in the doses of the poison, We
presume that if a few doses of the poison, in its less potent shape, were suffi-
cient to create typhus fever, a fortiori, such a quantity of it in a more concen-
trated form, as would be capable of producing a state of asphyxia not ultimately
fatal, would commonly at least leave the sufferer for days or weeks in the toils
of a highly dangerous fever, yet the reverse is the case, as the histories ot
mephitism amply demonstrate."
So they doubtless do, and they, moreover, shew that in some cases of recovery
from mephitism, a disease, apparently the effect of a morbid poison, followed*
though not fever. ,
But this writer has, like Dr. Smith, confounded the action of two poisons pj
different kinds?an inorganic poison, sulphuretted hydrogen, and a morbid
poison, whose action depends not upon its chemical qualities, but upon the
existing condition of its particles, they being at the time of their evolution in a
state of decomposition or transposition.
The advocate for the malarial origin of fever does not regard the fever poison
as the product of extreme putrefaction, capable of causing mephitism or fever*
according to the dose in which it is applied. But he holds that during the pr?~
gress to decay of organic substances, matter in a state of decomposition is
* "Dr. Smith illustrates and supports his doctrine of the malarial origin
fever by referring to facts which relate merely to periodic fevers ; and he main-
tains the identity of the ' fever poison' of this country with the poison of plague*
wherefore, on the principle, that things that are equal to the same thing afe
equal to one another?plague and ague are generated by the same poison !"""
Forbes's Review, No. 21, p. 13.
+ Vide Dr. Christison's article?Fever. Library of Medicine.
+ Forbes's Review, ut supra.
^41] On t/ie Poison of Fever. 39
it tn^e^LW^'c^ '3 caPable of communicating its state to the organism with which
ay .e brought into contact, while, on the completion of the process of de-
generat'ej011' ^ mor^'c' P?'son ceases to be evolved and the mephitic poison is
acc ^r'ect anal?gy to this is found in the effects of decayed sausages, which,
and r ^ to Christison, " are poisonous only at a particular stage of decay,
hvd Cease to so when putrefaction has advanced so far that sulphuretted
.r?Sen (the mephitic gas) is evolved." True, in mixed sources, and those
exi t ^fe receiving daily additions of new matters, the morbid poison may co-
eff ^e mephitic poison, and the latter may occasionally, by its sedative
areC s uPpn the nervous system, assist the operation of the former ; but they
essentially different in their nature and action.
Ken 0 Sec?ni^ objection?" an alleged want of proof that the fever poison is ever
of f6ra'"e^ 'n such sources"?rests, 1st, upon the relatively small number of cases
the fV6r S? Proc*uced, compared with the activity of contagion ; and, 2ndly, upon
are ac,:. ^at several diseases of different kinds, from tic-doloureux up to plague,
an to miasmic effluvia. Can we (it is asked) believe that they are
owing to the same poison ?
first of these grounds is urged against decomposing animal matters ; the
?nd chiefly against mixed sources, as sewers, banks of rivers, &c.
t is true that very few observations exist which can be said to prove the oc-
, rrence of fever from exposure to animal putrefaction?still, some such cases
C occurred. The following is referred to by Dr. Christison as an unexcep-
*"An American merchant-ship was lying at anchor in Wampoa road, sixteen
,lles from Canton. One of her crew died of dysentery. He was taken on
^ 0re to be buried; no disease of any kind had occurred in the ship from her
Parture from America till her arrival in the river Tigris. Four men accompa-
eu the corpse and two of them began to dig a grave. Unfortunately they lit
P?n a spot where a human body had been buried about two or three months
1 eviously, as was afterwards ascertained,?the instant the spade went through
0 ud of the coffin a most dreadful effluvium issued forth and the two men fell
wn nearly lifeless : it was with the greatest difficulty their companions could
Th r?ach near enough to drag them from the spot and fill up the place with earth,
fle two men now recovered a little, and, with assistance, reached the boat and
urned on board One of these men died on the evening of the fourth day,
nd the other on the morning of the fifth, after symptoms of malignant petechial
v?r (the petechise occurring on the fourth day).
" In eight days after the opening of the grave one of those who were not en-
&aged in the work was attacked with the same symptoms as his companions, and
le fourth had a slight indisposition of no very decided character."
It is to be remarked, that in the above case two circumstances were present
^hich we shall see have not always existed in the negative instances, brought
?rWard to prove that this is not a source of fever ; namely, " confinement of the
duvia," and a not very advanced stage of putrefaction. Ferriar, who did not
consider the exhalations from putrid animal matters a source of fever, says?
1 ' ^ appears from some late observations made on altering the vaults of a
ftUrch in France, that the confined effluvia of putrid bodies produce fever when
rought into action. Perhaps this is the solution of the question." J " Fourcroy
s ates, that the grave-diggers informed him that the putrid process disengages
Medico-Chirurgical Review, for Jan. 1825, p.
e^rs to the Mem. de la Soc. Royale de Med. 1. 97-
^ Medical Histories, vol. 1. On New Contagions.
+ Walker's gatherings from Grave-yards, p. 124.
203. Dr. Christison also
40 Extra-Limites. [Juty
clastic fluid, which inflates the abdomen and at last bursts it; that this even'
instantly causes vertigo, faintness and nausea in such persons as, unfortunate!) >
are within a certain distance of the spot where it happens, &c." In the exhu-
mations, conducted on such a large scale at the cemetery of the Innocents, an
quoted by Bancroft and others in proof of his position, neither of these con?'*
tions could have existed, since no interments had been allowed for six yearS
previously. .
In many of the cases also related by Mr. Walker it is mentioned that tn^
bodies had been buried for years, or were in an advanced stage of putrefaction,
under these circumstances mephitism was produced?but not fever.
The other part of this objection, namely that so many different diseases are
ascribed to this source?can they all be the effect of the same poison ? can onlj
be answered by supposing a variety of morbid poisons to be formed together or
consecutively in the same source. Several considerations render it probable
that this is the case in some malarial sources.
1. The progressive nature of the changes which the decomposing body under'
goes, and the different circumstances under which the same organic matters
(undergoing decomposition) may be placed in different places, or at different
times. There is nothing improbable in the supposition, that the same source
may at one time give origin to the poison of ague, and at another to the poison
of fever.
2. The fact that an individual exposed to these sources will frequently become
affected with two diseases. These will usually follow one another at a short
interval. " In the vast horde of cases," says Dr. Addison,* " which the river
side is continually sending forth, synochus and typhus are of frequent occur-
rence, and these are frequently followed, when the patient is convalescent, by
well-defined agues." Sir H. Marsh has noticed the same occurrence in the
epidemic of 1826, in Dublin, and it is well known that after this epidemic the
hospitals of that city were filled with cases of ague. This is perfectly analo-
gous to what happens from exposure to two morbid poisons, the one which ha3
the shortest latent period takes precedence and is followed by the other, as m
the case related by Dr. Williams of a boy who was inoculated at the same time
with the virus of measles and cow-pock. The cow-pock first ran its course, an"
was then followed by measles. In the same way it is very possible that the
poison of ague, imbibed at an early part of the year, may lie latent until the
conclusion of a continued fever received many weeks later.
3. This power of generating different diseases, is alleged of those sources in
particular which contain a variety of organic matters, and which are in a state
of constant change from the superaddition of new materials or from atmospheric
changes?such are sewers, the banks of rivers, &c.,?and it is to these that the
great body of evidence, as to the frequent production of fever, applies, and not
to the regular, uniform, and spontaneous decomposition of any single portion ot
animal matter, however great its bulk.f
* On Malaria. London Medical Gazette,?vol. 3, new series.
f If the subject admitted of an explanation purely hypothetical we mig^
draw an analogy, not destitute of plausibility, between the action of huma"
contagion and putrefaction in this particular ; and we might suppose, that as in
fever generated spontaneously in the human body, there does not seem to exist
any power of communication by infection, so in dead animal matter the product
of any single mass of spontaneous putrefaction is not a fever poison, but that
this is generated by the exposure of fresh dead organic matter to the contagion
of the former. For this hypothesis to be consistent with the facts, the follow-
ing should be the various consequences of exposure to putrefactive decomposi-
tion :?
^ On the Poison of Fever. 41
D
freq'CT" ?In order to obviate the objections urged against the evidence of the
c?nfoenti?CCU"ence ^ever fr?m malarial sources,?namely, that it has been
than ll" w'th contagion, and that, at all events, the evidence proves, no more
W t.^recluent co-existence of filth, fever, and poverty.
Unfav shaU select only a few cases which have occurred under circumstances
of ??'able to the supposition of such a cause as contagion, and the histories
particy] Present contrasts to that of contagious fever in some of the following
feVg'r ^class of persons affected, not those usually obnoxious to contagious
II Un'ess unc^er circumstances of prolonged exposure.
0j)n *.ccurring without the presence of any of the aids to contagion and at an
jyte season of the year.
jV* Realities in which contagious fever does not prevail.
t^ (j'ff P.reac^ng 'n sP'te of the preventive measures, which are found to check
"fusion of contagious fever.
? T
bet; n Sreat towns," says Christison, " cases are met with during the intervals
SeasoCetl epidemics, and in a station of life where epidemic fever in epidemic
tedi ns.?f the worst kind is seldom witnessed. A fever of this description,
Us ln its course, characterized by much nervous and muscular depression,
Ot any particular local disturbance, and, especially, without the marked
tVpi' r of the functions of the brain which distinguishes most cases of epidemic
of p"? and synochus, was so prevalent among the better ranks in certain streets
^0 ,. nburgh some years ago, at a time when fever was not prevalent among the
the ln^ classes, that a general impression arose among professional people of
Ujj fXlstence 0f some unusual local miasma. A great variety of parallel facts
'dent ^ re^erre(l to?all leading to the general conclusion, that a disease if not
ab0 ,lca^ w'th, at all events closely resembling, synochus and typhus as described
s'ck P' 111 arise without the possibility of tracing it to communication with the
v'sit' stateraent of this kind acquires great weight in the instance of such a
easv !on of disease as that just alluded to, which prevailed among people in
Y Clrcumstances in a great town."
apn ery similar is the testimony of Dr. Cheyne :?"For several years the fever
thevarec^ 'n families only in solitary instances, or if more than one were affected
t0 ^?^Vere seized nearly at the same time, but it did not extend so as to lead us
dise t'lat propagated itself. We were unable to assign the cause of the
lav ^urther than that we observed in several houses, in which our patients
sta * ^or which is discoverable when a sewer is choked, and, in some in-
b nc?s, upon enquiry it was found that the sewer leading from the house had
? ^properly constructed and neglected."
Un, Sl?ilar instance of fever, apparently caused by defective seweiage, came
Co er my observation recently in the house of a gentleman of fortune in this
Partly* ^or a l?n? time an unpleasant odour had been remarked in several
s ?f the mansion, more especially near this gentleman's study and in the
Du!" 3 rom exPosure to a single mass of animal matter undergoing spontaneous
Refaction?no fever.
(Jis * *r?m exposure to the emanations from substances added to the former
fav ase' varying in intensity in proportion as circumstances were more or less
Pos(Ura^e t0 rapid communication of the " contagion of decay" from decom-
^ng to recent organic matter.
tj0 " r?m successive exposure of a number of individuals to successive addi-
(Jig(;s organic matter, (under above circumstances), a number of cases of
42 Extra-Limites. *
men servants' sleeping apartment. The poisonous effects of the malaria
first produced in the form of obstinate dysentery in one of the female sen?wag
Then the owner of the mansion was attacked with what he at first supposed
mere biliary derangement, but which rapidly assumed all the characters of sev^
gastric fever, becoming attended toward the close with purple petechia and
minating fatally on the 11th day. ,er-
About the same time two men servants were seized with symptoms of ?eV .,
In one it was cut short and in the other it ran its course, ending faV0U^aes5
about the 11th day. Two other persons who came to the house on bus"? j
(from the neighbourhood) and who remained in it for a few hours, were se.).j1.
with the same fever, which ran through its course at their own houses butw
out extending to other individuals,
After this lamented occurrence the cause of the effluvium was searched
and found to be a leakage of the soil pipe of one of the water-closets, which
allowed the filth to percolate through the wall and exhale into the atmosplie
of the house. This exhalation was also much favoured by the warm tefflpc
ture kept up in the house by heated flues. . j
About the months of October and November, 1839,I was repeatedly consu1^
by the inmates of a large establishment in the neighbourhood of Navan, .
account of different forms of gastro-enteric affection, especially diarrhoea a
dysentery. So many instances occurred at intervals, (in some cases of wee* '
and the general resemblance was so great, that I thought they must arise ?
some local cause, and I expressed a strong suspicion that some source of 1139 fl(j
existed in the house or immediate vicinity. The house itself was large, airy*.9^
commodious, so that our inspection was directed rather to the immediate nel^,er
bourhood, and it was thought that the cause had been discovered in an old se^
which had been laid open in the course of some building operations. The clos'
of this was not, however, attended with the effect of stopping the endemic an
tion, though it gradually ceased after about a dozen people had been attac^
The following spring was remarkably dry, scarcely any rain having fallen
about six weeks ; toward the close of this period an effluvium of a very disagfe
able nature became perceptible in some parts of the house, and at the same tif"
?within a day of each other?two of the inmates were attacked with eXtlUhe
sitely marked typhus, attended with profuse measly eruption, and in one of of
patients with violent delirium. Every circumstance rendered the existence
contagion in either case highly improbable, I might almost say impossible,
on my again expressing my strong conviction that some form of malaria was
cause of the fever, I was informed of the effluvium perceived in some of
passages, and also of the fact that in the original construction of the wat
closets they had been made to depend for their supply of water upon a c'steQt
of rain water, which, of course, had been for weeks empty during the PreS?n,
spring and preceding autumn. These cases did not spread, and all traces of 1
disposition were removed by making the required alterations for ensuring aC?
stant supply of water. ^
In the month of October, 1839, I attended a respectable man who resided
a large and airy mansion, as " care taker," during the absence of the fa?1 J
upon the Continent. His illness had come on slowly and insidiously, but, ^
I saw him, had all the characters of bad remittent fever, attended with i?uC.t
abdominal congestion. This it was attempted to relieve by leeches, &c., buL?
increased and led afterwards to large evacuations of blood from the bowels, r
recovered slowly and with difficulty. At the time I saw him he was ly'11?
the basement story of the house. . ^
A few weeks after the return home of the family, the butler was attacked a
symptoms of gastro-enterite and slight jaundice. He recovered partially
few days, but in the course of a week after was suddenly seized with comp1^
loss of muscular power, paleness and coldness of the surface, sickness
On the Poison of Fever. 43
1841 j
lar^g^' ^c"'. followed by vomiting of a dark olive fluid, and in two days by
the SKGVacuat'ons of tarry blood from the bowels, hiccup, subsultus, &c., while
?f larc" covered with vibices and black petechia?some of them of the size
fectlvge s"ot- The fever which followed had no perceptible remissions and per-
othersaC<TIC'e^ t'le descriptions putrid malignant fever by Huxham and
WhichV striking resemblance of some of the symptoms of this case to that
bute 1 occurred in the same place more than a year before, led me to attri-
f?llow *? a coramon cause and to enquire for the source of the malaria. The
pi6(j , ln? were the facts ascertained :?In a room in the basement story, occu-
ert>Pt ' 6 ^ast Pat'ent and in which he had latterly sometimes slept, was a sink
, which communicated at the distance of about ten feet with
passed*"* sewer of the house?into which the contents of two water-closets
fr0m t]" ^ his sewer was very large at its termination and when the wind blew
the ^'rect'on towards the house, there being no smell trap under the sink,
p?rtahiUVlLm ?*" sewer was cairied up into this room and became so insup-
retirino.e the patient used to stuff the aperture with a piece of rag when
quenti^ k? ^e(^' ^Pon enquiry I was informed that the first patient had fre-
reniarlJ , ore his illness remarked the same fetid effluvium. It is worthy of
Th V the sewer had not been cleaned in the interval between the two cases,
the n ..wing case, very similar to the last in its nature and origin, I give upon
stro,*^ authority of an anticontagionist* :?" I attended," says Dr. Arm-
of tv8't "a very respectable tradesman, labouring under a remarkable bad attack
of ? j s fever. It was such a case as would have been called plague in the time
Was ^ nham. He had knotted glands and carbuncles, and black petechise. He
i}jan,0ne. ?f four or five individuals who had transacted some business inanoble-
the fS tclien ; a filthy fluid had overflowed that kitchen ; he was sickened at
jj.ltne. and in common with all the others had an attack of typhus fever."
we ^e,'ooked about for a large town less liable to contagious fever than others
Which n Pr?bably find it in Birmingham,?yet here endemial causes of the kind,
feVtr ~!r* Davidson has pronounced inadequate to this effect, have produced
endel- good instance for illustration is found in Dr. Ward's account of an
of fever, which prevailed in certain localities in Birmingham in the summer
t"Th ?
serVes river Rea> that separates Birmingham from its suburb Badesley and
turn as a cloaca maxima to both, carries its filthy stream onward, partly to
last v In'" anc* Part'y AH a ra'H pond. During the drought which prevailed
Hot ?ear the water was very low in the main stream and mill pond, and the mills
strea e'n^ regularly worked became quite stagnant and offensive. The back
Wett U) a'so became dry and shewed its mud banks, that were only occasionally
surD| hy a flush of the washings of the town after a shower, or by the small
exhaiUs. accumulated during the cessation of the working of the mills. The
nig. atl0ns from the half dried mud and putrid water were so disagreeable at
s0on as to nauseate the more delicate inhabitants of the adjoining streets, and
H7 produce disease in the form of typhoid fever of an infectious (?) character."
vicj^Pes ?n to state that about 50 cases?some fatal?occurred in the immediate
as y ?f the stream, and "still lower down the stream, where the water was
pail c* as ink, there were 13 pauper cases in one yard, and many others, both
8^^ rfanc* Private, along the same line." That this fever was owing to the
the stream is proved by the disease being confined to the locality, the
the v numher affected in so large a population as Birmingham, the season of
-^thV,ear' anc* the exemption of this town from the causes which aid contagion
Se are well-summed up by Dr. Ward.
See Christison, ut supra. -f- Lectures by Rix.
i Provincial Medical Transactions, vol. 6.
44 Extra-Limites. [Ju^ '
" There is a difference of nearly 200 feet in the elevation of different parts of ^
town. The streets and the courts or yards in which the mechanics live are ,
and airy in general; fuel is cheaper than in any other large town in Eng'a /
the water is excellent?and till within the last year there has been but 1'
distress." . tj,e
We have already adduced the effects of seclusion of the sick in proof
infectiousness of typhus. In the fever arising from endemial sources this ?
sure has no such influence. I was much struck with this fact when ma' ?
some investigations as to the source of a fever, which prevailed in the sum .
of 1839 in a hamlet attached to a flax manufactory near this town, from
a considerable number of cases had been sent to hospital in the months of AP '
May, and June. The object of an examination which I made of the place v
to obtain satisfactory instances of contagion, but I soon found that no suche ^
dence was to be procured. For the intervals between the illness of differ
members of the families were too irregular to admit of communication fr0.?0^
to another. Thus, in one house the first case sickened on the 2nd of April' a
the second on the 5th. In another, more than three months intervened betwc
the first and second cases. And in several families in which the first case I1
been early removed to hospital, the second had sickened before the patieI^
return. Besides, there was too much cleanliness and comfort : several of
houses had been repeatedly white-washed during the time that the fever J'
going through the family, and the inmates were all well off?being employe11
the neighbouring factory. jet
Several things convinced me that this fever had a malarial origin. The ha"1
was built in the form of two parallel streets, terminating in a large open sP.ano
in front of which were twelve houses looking North East. This space ha'a
drainage and was full of shallow pools of black putrescent water, into which
inmates daily threw cabbage leaves, &c. to rot for manure. In this country ^
East and North East winds prevail for the first three months of summer?AP ^
to June?and in consequence the inhabitants of the twelve houses descri
were peculiarly obnoxious to the emanations from these pools. The weatn
had during the summer been unusually dry and favourable to such emanatio0^
Accordingly I found that while only seven cases of fever had occurred in twee*)
two houses, forming the longer of the two streets, 30 out of 50 (the ent'r
number) had occurred in these twelve houses. , e
The proof was to my mind rendered complete by the immediate effect of e
heavy rains which set in in July. The disease was stopped at once and I 'ia
not heard of a case of fever in the same place since. _ ^
Similar proof, derived from the sanatory effect of the removal of the assign
cause, was afforded me in the case of a house in this county from which ^
servants were sent into the Navan hospital at short intervals labouring u0 .
continued fever, one of whom was also admitted a second time with sev'e
dysentery. A very offensive smell had been long noticed in the yard adjoi?'?
the kitchen, and after the occurrence of these cases the sewer leading from* j
kitchen was in consequence examined, and found to be completely obstruct
by a quantity of black putrescent matter. Upon the removal of this the stf?
of course disappeared and no return of indisposition has since occurred, _
among the servants or family. There was not the slightest evidence of coo'a
gion in any of these cases. , , ^
A reason for attributing the fever to the operation of endemial causes W'S g
be found in some instances, in the fact of the great indisposition of the disc^
to spread in the house in which a case occurs, even though the circumstances > ?
vourable to contagion may be present. Such an instance is given by Dr. Fe
gusson which we shall have again to notice. ( e
In a paper on the statistics of fever, in Belfast, Dr. Mateer states that " 0?
street, Carricks Hill, and its continuation, Mill-field, with the adjoining la?
1841]
On the Poison of Fever. 45
*n<1 entries, are found to have furnished three fifths of the whole amount of
ases, and yet they are by no means the poorest or worst ventilated parts of the
fje attributes the prevalence of fever in this locality to the great want
Water?" the consequences of which are want of cleanliness and bad sewer-
~e' So that decayed animal and vegetable matter of all kinds, not being cariied
j. . a current of water in the usual way, accumulate and generate mias-
mata"* This obscrvation ;3 0f the more value that Dr. Mateer adduces it in
,,?Pport of the action of infection. Upon which an acute reviewer remarks
of lJre'y this offers the very strongest argument against Dr. Mateer s own view
* the ? ?
Part* ?xtens*ve operation of contagion ; why should this be all powerful in one
l'lat'CU ^oca'ity ? why should it not do its worst where poverty and bad ven-
ftiore?^ ^0Ur'sh ? why, but that on the large scale other causes of fever are far
worst where poverty and bad ven-
on the large
^ potent than contagion." f
ral ? t^le following case by Dr. Currie illustrates this form of fever in seve-
,^'ticulars :?
t?Wtl ^Oth Regiment, as is usual with troops in Liverpool, was billetted in the
1 ut paraded and mounted guard in the fort, situated north of the town
t0 tj?n the banks of the river. The general guard room had been used, previous
tre e.arrival of the 30th, as a place of confinement for deserters; it was ex-
full f c'ose and dirty, and under it was a cellar, which in the winter had been
offD ?. w&ter. This water was now half evaporated and from the surface issued
j iSll'e exhalations.
co fl a dark' narrow, and unventilated cell off the guard room it was usual to
be ? ne. such men as were sent to the guard for misbehaviour, and about the
of inning of June, 1792, several men had been shut up in this place on account
Jit runkenness, and suffered to remain there twenty-four hours under the debi-
t\v0 succeeds intoxication. The typhus or jail fever made its appearance in
Ten t^ese men about the first of the month and spread with great rapidity,
pof the soldiers labouring under this epidemic were received into the Liver-
c&Se t^firmary The symptoms of the fever were very uniform, in every
who there was more or less cough with mucous expectoration ; in all those
the ?ustained the disease eight days and upwards there were petechise on
bio i ?' *n several there were occasional bleedings from the nose and streaks of
G ln the expectoration. The debility was considerable from the first
the 'n the head with stupor pervaded the whole, and in several instances
to m-6 0ccurred a considerable degree of low delirium Our next care was
first Pro?ress ?f the infection ; with this view the guard house was at
it f attempted to be purified by washing and ventilation ; the greatest part of
ex urn'ture having been burnt or thrown into the sea. All our precautions and
at tr?n? this kind were, however, found to be ineffectual. The weather was
lot K t'me Wet' anc* extremely cold for the season ; the men on guard could
inf Prevailed on to remain in the open air and from passing the night in the
Ij ^ted guard room, several of the privates of the successive reliefs on the 10th,
f0 ' and 12th of the month, caught the infection No means having been
por e^ectual for the purification of the guard room it was shut up, and a tem-
?f S^ed erected it its stead. Still the contagion proceeded on the morning
dav V^h' three more having been added to the list of the infected. On that
exa' ? eref?re> the whole regiment was drawn up at my request, and the men
the 'n their ranks : seventeen were found with symptoms of fever upon
Tjj ? ' ^ was not difficult to distinguish them as they stood by their fellows.
Hiat" Countenances were languid, their whole appearance dejected, and the ad-
a ?f their eyes had a dull red suffusion. These men were carefully sepa-
* Dublin Medical Journal, September, 1836.
t Medico-Chirurgical Review, Oct. 183G.
46 Extra-Limites.
rated from the rest of the corps and immediately subjected to the cold a^s!C"f
.. ..These means were successful in arresting the epidemic?after the l->
June no person was attacked by it." .ed
It may seem presumptuous to ofTer an opinion differing from that exP*,e^ut
by the distinguished writer under whose observation these cases occurred, ^
we think there is every reason to question the existence of infection, an ^
regard them as of purely endemial origin. Let us consider them with reteren0^
the circumstances unfavourable to the existence of contagion before enurnera^
1. The class of j)ersons affected. British soldiers in time of peace are not
noxious to contagious fever. The fact is stated by Dr. Cheyne, that while'e ^
of this kind prevailed in the street contiguous to the principal barrack in V ,
lin, in 1817, and among a class of persons with whom the soldiers comW? -
associate, they escaped, because "little under the influence of the predisp?s ,|
causes of fever; for the pay of the soldier is ample, he is well clothed,
fed, well lodged, and well looked after, and all his wants in health as well
sickness are provided for." * , j5
2. The season of its occurrence is another strong reason for considering
fever of an endemial kind. A contagious epidemic may live out the sum0'1'
but unless it is imported we should doubt its being generated at that season-
3. The locality was also unfavourable. Isolated as it was, an imported con
gion was unlikely. j0<
4. The inefficacy of all the preventive measures, short of removal from the ^
cality, with the immediate cessation of the disease which followed this step,
strongly opposed to the idea of infection. In fact if it be admitted that
stage of maturation or crisis is the period of infection, an examination of
dates of these cases will shew that in no one was the disease so far advance
to have enabled the patient to communicate it to his comrades, supposing tfl .
(which is not at all probable) to have had access to the hospital. On the ot ^
hand the positive evidence in favour of malaria is clear and decisive. Several
dividuals were exposed to this source during the debility which succeeds ii't? .
cation, and slept in its immediate neighbourhood. They were attacked, and jS
in succession as they became exposed to the same source. The malarious sP?^.e
abandoned on the 12th, and no case is observed after the 13th. Hereafter |
shall attempt to shew that the symptoms of these cases were such as charac
rise not typhus but typhoid fever?especially the late appearance of pete 0f
the exudations of blood from the air-passages, and the form of disturbaoce
the sensorial functions.
The above are a few of the instances which might be brought forward ^
prove the occurrence of fever in situations and circumstances unfavourable .
contagion, and not liable to the objection that filth has existed merely in fort
tous connexion with fever and poverty. It would not be difficult to draw fr
the published histories of infectious fever (so called) such a number of si#' ^
facts, as would render doubtful the justice of Christison's objection, that '
for the few instances iemaining, where true primary fever appears to orig'n'
in one of the above causes, all that need be said farther is that for one iiistao
where such fever follows such cause, a thousand instances occur where no en
of the kind ensues, and that, consequently, some more essential influence is P
bably brought into play, than what appears merely on the surface of the 'nVejt
tigation." But some argue that the disease produced is not fever?for, ^TS\eC
does not diffuse itself as fever does by infection. This is not the place to eD
upon an examination of the conflicting statements upon this question-^
shall do so hereafter; but admitting that it has been asserted too hastily by ^
S. Smith, and others, that infectious fever is generated by paludal sources,
Dublin Hospital Reports. Vol. 2.
18411
J On the Poison of Fever. A*]
""el ^at t'^'s J'ust'fies the inference sought to be deduced, that, therefore, conti-
reasoV?^-1S no1' so gene,,ated. On the contrary, it seems more consonant with
^itho, ? ln^er> that if fever affects a number of individuals in a certain locality
Mr'tho' ^PPearing to be communicated from one individual to another, and
fr0m J ' ln any instance, being carried from that locality, this fever must arise
are in ?me 'oca^ s?urce common to all the affected persons. And if that party
generafXIreme' wh? hold that fever of a contagious specific character is daily
Posite ^ common causes external to the human body, equally so are the op-
Whiig party who deny to these sources the power to cause fever, " not typhoid,"
cific a*" *he same time they are ready to admit the identity of their own " spe-
Dever ?nta^'0n" a ^'sease which, the most eminent observers maintain, is
alih, Cf?n^aS'ous! It is surely more consistent with the doctrine of the speci-
tlQUcd f ^ P^us to 'et it stand alone, and to give a place to non-contagious con-
typh0- ie/er' than to exclude the latter by a doubtful assimilation of typhus and
is ju c evei's. Hereafter we shall attempt to shew that the most recent science
'typhaCC'?r<ianCe Practical observation of Grant, that "these fevers,
their US' ai? generally contagious which the common fevers are not, unless
We "ature is altered, and they are rendered malignant by bad treatment"?while
\vi(]el J See reason in the present state of society in our large cities, in the
Cons Preva'ling influence of crowding, poverty, non-ventilation, &c., and the
the^ntly frequent and facile transition of common into contagious fever, why
Why .??t opposite conclusions are formed as to their origin and diffusion, and
the ?r aPPens, as Christison truly remarks, that " the greater proportion of
ess '?crePant doctrines of the present day as to the origin of fever are founded
'ally upon the same great body of facts."
digv^ain> by some it is urged that the disease produced by paludal emanations
a||e?rs fr?ni continued fever in symptoms and in type. Thus Dr. Christison
titiu | l^a* " ^ew inquirers have taken sufficient pains to distinguish primary con-
haVt; ^ever from irritative gastric fever." This objection cannot be allowed to
(liS[} ^Uch weight so long as the primary nature of typhoid fever is a matter of
his n 6" ^Pon the subject of the type Dr. Christison may be quoted against
tropj!"}'' if, as he asserts, " The coast remittent fever of Africa and other
*aPid C0Untries seems to differ little in its characters from synochus, with a
aga; an<^ early stage of typhoid depression,"* what becomes of the argument
the malarial origin of continued fevers from alleged differences in the
^ ?f these and the intermittent and remittent fevers, also produced by
giotjj,!a' besides how can the exclusive contagionist answer the anti-conta-
"who rests his doctrine on such facts as those adduced by Armstrong:
stren?rtly after * ^ad published my 3rd edition on typhus fever, in which I had
interU?Us,y maintained the doctrine of human contagion, I met with a case of
ftior fever; in a few days the fever became remittent, and in a few days
?Half I>Ut 00 the continued character, and the patient died with all the most
s?n ??ant symptoms ?"+ or how will he dispose of the assertion of Dr. Elliot-
hat most cases of so called typhus fever are really remittent, J or explain
+ r * Library of Medicine. t Lectures by Rix.
D^+wh^'Jres ^ R?gers> Dr. Mateer also observes?" We have the paroxysms
but sf'ii ^ever's made up best seen in the intermittent and remittent fevers,
Hiask i ^ careful observation we can detect something of the same kind, though
These i&n^ ?^ten difficult to recognize, in the continued fevers of this country.
Joiirn a niost always assume more or less of the remittent character."?Dublin
br A ut suPra-
?bser" \Urr'e remarks, whoever has watched the progress of fever must have
oiberV the justness observations made by Cullen, Vogel, DeHaen, and
s' ^at even those genera which are denominated continued are not strictly
48 Extua-Limitks. '
the occurrence (already noticed) described by Marsh and Addison of
ague, following on the subsidence of continued typhus or synochus ? ^as ^
ague also the effect of contagion ? Or will the contagionist escape fr?nl
necessity of adopting so easy a solution of the difficulty as the supposit'0"
different morbid poisons, generated at different periods in the same locality )
simple denial of the fact, and an impeachment of the accuracy of the ?')sero,vca
who have recorded it ? " In Sydenham's time," says Dr. Hancock, " and e
in that of Fothergill, the quotidian of spring became continued fever in sUII\ ,0g
while the simple continued fever of summer often changed to a malignant )Y
in autumn. These were simple observations at a time when systematic ai'iaD.?
ments had not put physicians in trammels. But now lest we should be gu'^j
of medical heresy we must not insinuate that ague can change into con.tl"jial
fever, and non-contagious fever into contagious typhus, either in an indivia
case or in the course of the year."
Sect. III.?Varieties of the Sources and Modes of Application of the Pois?r"
The organic matter constituting the source of the morbid poison may bepurC'-
animal, vegetable, or a mixture of both. re
It must be admitted that fever seems to be very rarely produced by exp??
to purely animal exhalations, and the facts brought forward by Bancroft, y
holm, Duchatelet, and others, shew that in the great majority of cases this
posure has been continued for any length of time with perfect impunity* ^
still there have occurred well authenticated instances to the contrary, son1?
which have been referred to ; and a fact lately published, by M. Devergie, dese1"^
farther notice. It is the occurrence of hospital gangrene in the hospital of
Louis, which he attributes to the emanations from Montfaugon, since the dise ^
was confined to the wards which were exposed to those emanations, and did11
appear in other parts of the building. u
Now if we admit the inference which seems naturally to follow from sj\.
instances as those related by Pringle, Hennen, &c., of the occurrence of tyP^ .
in the unwounded in wards, in which hospital gangrene existed, and of tVp^
attacking the attendants employed in washing the bandages of the sarD
namely, that hospital gangrene is a modification, or as it has been expi"esse '
" a visible personification" of the typhus-poison ; we cannot avoid the
sion that a fever poison may be generated by decomposing animal matter ud
certain conditions. ^
What the conditions required for this result may be, and why it so se' J
happens that fever is thus produced, are questions to be resolved by deeper 8
more accurate investigations than appear yet to have been made. . ^
There seems to be a more general belief in the activity of the vegetable P015^
though why it might be difficult to say, unless from juxta-position with ^
known fact of their power to cause periodic fevers, since there is at lea5l:re,
equal paucity of strict evidence with regard to this as to the animal s0"rver
About 15 years since I witnessed the origin of a highly typhoid petechial fe^e
in a healthy village in England, which appeared to arise from a vegetable sour
acl*
such, but have pretty regular and distinct exacerbations and remissions in e
diurnal period.?Med. Reports, p. 16. j
And Dr. Fordyce, says the similarity between these three kinds has deteriB'.1^
practitioners of the greatest eminence, through the whole history of med'C i
to consider them as the same disease. Many have thought that in a contin ^
fever the subsequent paroxysm takes place in the hot fit of the prior parody9
&c.?(3rd Dissertation, p. 59.)
On the Poison of Fever. 49
1841]
^ r\f*
tvv? or tVi PUtrel>inS turnips- In a bouse close to the nuisance, a boy had for
^ not lFee Wee^s keen complaining of headache, lassitude, and debility, hut
he ha[j u een P^aced under any medical care. On the day on which I saw him
followi Cen packed w'th epistaxis which continued till his death, on the day
death d"?' ,His s^'n was covered w'lh small ecchymotic petechiae. After hi'3
adi -C ^6Ver aPPearec* ^ the family, consisting of six persons, and in
bey0nj'?lnS houses, and proved fatal in several instances. It did not spread
(sunjm l locahty and subsided in a few weeks. The season of the year
of the other circumstances, were unfavourable to the supposition
g^tagion.
all oh 11 'S t0 sources containing mixed organic matters that the experience of
slaug^,. ryeis P?int as most efficient in producing continued fever; such arc
t? t[le erh?uses, obstructed sewers, cesspools, &c. &c. It were needless to add
Thg eta'is already given of cases originating in these sources.
^trodu^- S- 'n w^'c^ ^e poison may be applied to the organism are by direct
aid lixr ?i'?n 'nto the circulation, by being taken into the stomach, by inhalation,
p, uy the skin.
r^VPr
produ ' ?.r a disease confessedly bearing a close resemblance to it, has been
dwell 10 t^le 'ower animals by experiments too well known to need us to
diseasUP?n them, and occasionally the typhoid symptoms which appear in other
circu[ s. would seem to be owing to the absorption of putrid matters into the
b?yao. lon- A case of the kind once occurred under my own observation: a
fever h ten years was received into hospital, labouring apparently under typhoid
\vas 'a sunk after a few days, and on dissection the only lesion discoverable
Vritjj cai''ous state of the petrous bone, with a minute opening communicating
pas?n,le lateral sinus, through which the matter of the carious abscess had
j into the circulation.
talcens- rarely that an instance occurs of fever produced by putrid matters
Up0n lnto the stomach, and the immunity enjoyed by savages, who live much
a&ains'tUt|!^ ^es^' ^c'' ^ias ^een relerred to by Bancroft and others, not only
tainjnn. e fact* but also against the supposition of putrid animal matters con-
case a ^ever poison. It is not difficult to understand that this should be the
rertja t'n.ce digestion, an antiseptic process, precedes assimilation, and changes
by r: ^ y the matters submitted to its operation ; or, as accurately expressed
acids C Putrid poisons having an ulkaline reaction are rendered inert by the
saySa^0Rta'ned in the stomach, while these exert no such power over poisonous
ti0tls ges which have an acid reaction. But this rule is not without someexcep-
in ?/ Dr. Christison's work on poisons is a report of a case which occurred
withh ?rt in the year 1830, of a family of five persons who were poisoned
sever ^ made of putrid beef ; in three instances the disease produced was
iu0(j C' anc* in one fatal. It is worthy of remark (and is in accordance with the
c0me ot action of a morbid poison) that in the worst cases the illness did not
Casemence till the second, and (in the fatal case) the third day after the meal. A
in Is s?mewhere narrated of a regiment in which putrid fever prevailed, and
uSed Ich the disease was checked upon a discovery being made that the water
stat0 ?J ^'inking was drawn from a well in which some bodies were lying in a
^of putrefaction.
Paris' P'and* remarks upon the effects of drinking the water of the Seine at
Water an^ "^r" Uncock observes, that it has been frequently remarked that this
it. 4, Pr?duces diarrhoea in every one except the Parisian accustomed to the use of
of (jr- , f* Tweedie refers to the history of a fever ascribed to ihe combined effect
'nking putrid water and the emanations from the same?and others ascribe
* Dictionary of Practical Medicine, art. Endemic Influences,
t Cyclopaedia of Practical Medicine, art. Endemic Diseases.
D
50 Extra-Limites. [Ju^
the putrid fevers of Paris to the fact that " there are numerous wells in that city
from which many of the inhabitants derive their whole supply of water, n ^
few of which are situated in the very neighbourhood where the ' fosses' are ? ?
worst constructed and the least attended to ; the urine, therefore, pernaea ^
the soil must necessarily contaminate the springs from which these wells
fed."* j_
The evidence given before the committee on the health of towns, by ^ 'go
B. Wood, bears upon this question. After stating (qs. 2150-4) that 31,
persons live in the cellars of Liverpool?forming two-thirds of the wor? =
population?he states that, " in the districts in which these cellars are situa '
there is a great deal of broken ground in which there are pits ; the water ac
mulates in these pits, and of course at the fall of the year there is a good
of water in them, in which there have been thrown dead dogs and cats an
great many offensive articles. This water is nevertheless used for culinary P' ^
poses. I could not believe this at first, I thought it was only used for washl0f'
but I found it was used by the poorer inhabitants for culinary purposes." , . ?
The change produced in the fluids and solids of an animal by over drlV',?
seems to be capable of becoming a cause of disease in the human body. *D
remarkable case given by Andral from Du Hamel it does not appear that fe; \
strictly speaking, was produced. The effect rather resembled hospital gangrct1^
but an instance is recorded of typhous fever following from eating the fleS^( ^
animals under similar circumstances. It is thus quoted by Dr. Gross : t
few years ago a number of fattened cattle were driven into one of the New
land cities, and having been pressed too hard in a sultry day were sooverhea^
that some of them became quite exhausted. In this condition they were slaug
tered, and the consequence was, as is stated by the reporter of the case, V ^
Fountain, that nearly all who partook of their flesh were seized with typb?
fever."
These and similar observations would seem to shew that the morbid P?lS?J
the product of putrefactive decomposition, may be received into the sVste
through the stomach, more especially if presented in the fluid form ; but tne
is every reason to conclude that fever is but seldom produced in this way, an3
that the general mode of introduction is through the respiration of the gase?Uj
exhalations, which, as we have seen, are found upon their being collected an
condensed to contain animalized matter in the state of progress to decay, wh?5^
power of producing disease, in those exposed to their influence, has been q?eS'
tioned and is by many denied, but appears to be proved by evidence of a ve. ^
satisfactory nature, and which applies with most force to cases occurring un"
circumstances unfavourable to the action of contagion.
Sect. IV.?On the Mode of Action of the Poison.
This does not appear to be attributable to its chemical qualities, but to rtsC??t
dition. It is the power of communicating an action, since there can be no dou
that its effects continue to be produced equally after the removal as during ^
presence of the cause, when once this cause has impressed its mode of aC^?e
upon the organism. What the mode of this impression is, and what part 0
system is the subject of it in the first instance, we have now to enquire, as 8'?
into the order of the phenomena subsequently produced and constituting *
formed disease.
The generally received opinion seems to be that the nervous system is
* Medico-Chirurg. Review, vol. 6.
f Pathological Anatomy, vol. 1, p. 223.
the
^ On the Poison of Fever. 51
Sorbin mork?fic impression, and its derangements the first in the
Th' ^erie? constituting fever.
dia(.g1S . ^rine is thus maintained by Dr. Southwood Smith*?"Theimme-
Up0n i?c,''nS cause of fever is a poison which operates primarily and specifically
are i le brain and spinal chord. The diseased state into which these organs
iHUnj 0u.Sht by the operation of the poison, deprives them of the power of com-
ence-,CatlnS to the system that supply of stimulus, (nervous and sensorial influ-
of jj ' ^'hich is requisite to maintain the functions of the economy in the state
lerv ? orSans? the seats of the functions, deprived of their supply of
a fjx?jS ^Anence, become deranged, the derangement in each taking place in
Sub ?rc*er an(* *n a determinate manner.
are Seciuently to the nervous and the sensorial, the organs the next to suffer
*hic,f ?f the circulation, then those of respiration, and, ultimately, those
Which nS to secretion and excretion. The condition of the nervous system,
thaj Produces this derangement in this circle of organs, occasions further, in
vess P?ftion of the circulating system which consists of the capillary blood-
is a|6 S' peculiar state which constitutes inflammation : hence inflammation
kbril ?S-^ alwa}'s established in one or more of the organs comprehended in the
Wr|t 6 Clrde and sometimes in all of them." In another passage the same
says?"The more closely and extensively the subject is investigated the
of f c,ear and satisfactory the evidence becomes, that the great primary cause
the eVer 'S a P?'son> the operation of which, like that of some other poisons,
Co H^ture of which is better understood and the action of which has been more
poi ^ 'y examined, is ascertained to be upon the nervous system. How these
!*>?? act upon the nervous system we do not know, nor can we possibly
?f aS as we remain so profoundly ignorant of the nature of the action
j e nervous system in the state of health."
am VVl" seen that Dr. Smith's argument, like that of others already ex-
the ? 's rested upon a fallacious analogy to certain other poisons, and like
Up e !s open to the fatal objection, that such poisons really produce their effect
1^0^ nervous system subsequently to their diffusion through the blood. It
a n f?Ver tacks the support derived in the former cases from the occurrence of
s0UrrV?US s^ock' since such rarely, if ever, attends exposure to the paludal
hu Ces fever. On the other hand, some of the arguments for a modified
Uj- ?rai theory before adduced, apply with greater force to this than the animal
d Stn' and additional ones are not wanting to strengthen the proofs that all
Poi nSe.ments of function in fever are subsequent to the introduction of the
ter)Son.'nt? the blood, and the consequent vitiation of that fluid. For, its la-
cy 's even more remarkable than that of the animal infection of typhus,
"e a close observation of the state of the patient, during this period, will
a derangement of the secreting and excreting functions, which seem to be
te ^Ur'ng to rid the system of the poison, or of those products which it has a
au ency to generate in the blood ; again, we can not unfrequently trace the
rupt termination of this period, and the supervention of formed disease to a
Pension of the depuratorv action, by cold, intemperance, or any other cause
of n, ^tnrbs the order of the excreting functions and arrests the elimination
?Wh if morbid product. Thus, in an exquisitely marked case of paludal fever,
lcn was lately under my care and which terminated fatally after large evacu-
fri blood from the bowels, it was remarked spontaneously by the patient's
fro S ^at, for six weeks before fever commenced, not only had he suffered
Co ^ caPricious appetite and irregular bowels, but that a remarkable thick
he^]?US deposit had been constantljr present in his urine. During this period
lad been living and sleeping in immediate proximity to a collection of filth,
* Treatise on Fever.
52 Extra-Li mites. [July
which at times filled the house (in other respects a cleanly and comfortable
with an insufferable putrid effluvium. . , js
This instance also illustrates the cumulative property of the poison, wh'C
much more remarkable than that of the animal infection. Unlike the 1^ '
which is often most severe upon its first introduction into a family, the p01?
of malaria seems commonly to affect each successive patient more severely 111
the preceding.
To what can this be attributed but to the accumulation in the blood c?nle
quent upon longer continued imbibition of the poison ? This is similar to
explanation offered by Cruveilhier, of the general incurability of phlebitis from j
absorption of pus. Experiments have shewn that, from a single injection of pu
pus into the vein, an animal may, after copious evacuations, recover ; he there!
concludes that similar success might attend the evacuant treatment of phlebi -j,
did not the renewal of the sources of infection follow the incessant renewa
the pus. But we can not only detect the presence and agency of the P01??n.i,e
the circulation through the deranged excretions, but also occasionally 'n
physical changes of the blood itself previous to the occurrence of formed
ease. The following observations of Dr. Potter* on this subject, are highl)'10f
teresting and important, and from the care and accuracy with which they a?Pe^e
to have been made, are entitled to great weight in the discussion of the
of access of fevers arising from malaria. He says, " it was remarkable
all cases in which it was deemed expedient to bleed, the blood wore the sa"
general appearances. After a separation had taken place, the serum assume ^
yellow shade : often a deep orange, and a portion of the red globules was i&va
riably precipitated.
It occurred to me that if the remote cause resided in a common atmosp''e '
the blood of all who had inhaled it a certain time would exhibit similar P^eD^0
mena. It accorded with the pathology I had conceived, to conclude, that all ?
lived in an atmosphere so impregnated were constantly predisposed, and thata
additional or exciting cause only would be required to develope the symptoI.n
and form. To ascertain the appearances of the blood in subjects apparently 1
good health, I drew it from five persons who had lived during the whole seaso
in the most infected parts of the city, and who were, to every external aPPea[j
ance and inward feeling, in perfect health. The appearance of the blood cou
not be distinguished from that of those who laboured under the most invetera
grades of the disease. As this experiment might have been considered in???a
elusive unless the blood could be compared with that of those who lived in .
purer atmosphere, remote from the evolution of miasmata, I selected an equ
number of persons who lived on the hills of Baltimore County, and drew fr?
them ten ounces of blood. The contrast in the appearances was so mani'1^
that no cause for hesitation remained. There was neither a preternaturav
yellow serum, nor a red precipitate ; the appearances were such as we
the blood of healthy subjects. A young gentleman, having returned from "i
western part of Pennsylvania on the 10th of September, I drew a few ounces 0
blood from a vein on that day ; it discovered no deviation from that of ot'ie
healthy persons. He remained in my family till the 26th of the month, and0
that day I repeated the bloodletting. The serum had assumed a deep ye^?i]e
hue, and a copious precipitate of red globules had fallen to the bottom of ^
* Quoted by Dr. Tvveedie, Art. Fever, Cyclopaedia of Practical Medicine.
Dr. Tweedie's own expressed opinion is " whether the opinion of the olde
writers, that in fevers originating from contagion, the contagious principle alter6
the properties of the blood be correct or not, we certainly think the strong a"3"
logy in the cases alluded to tends to confirm the supposition of typhoid feve'5
originating in diseased blood."
On the Poison of Fever. 53
y I r\
the r vessel- Of the six persons whose blood assumed those indications of
eSc er^?te cause, four were seized with fever during the epidemic ; the other two
?thp an-T ^ormal attack, but complained occasionally of headache, nausea, and
r ,r>dications of disease."
the experiments were instituted by Dr. Stevens, and with a like result,
to th being black in colour, and evidently deranged in its properties previous
e commencement of the fever.
, ^'ght almost be considered unnecessary to strengthen the inference from
eVo] ac'ts as these, by observing on the effect upon the blood of the gases,
Co v. from sources which we consider the evidence already adduced proves to
Ce ain. a fever poison, when these are presented in a sufficient degree of con-
^ration to produce rapidly fatal effects.
escribing the consequences of exposure to the emanations from Parisian
by ,1?S' Dr. Christison says :?"The appearances in the bodies of persons killed
int emanations are fluidity and blackness of the blood, a dark tint of all the
0r ifna^ vascular organs, annihilation of the contractility of the muscles, more
,ess redness of the bronchial tubes and secretion of broivn mucus there as well
lik"1./ n?strils, gorging of the lungs, an odour throughout the whole viscera,
\V decayed fish, and a tendency to early putrefaction."
cj . 'th these and similar facts before us, we cannot agree in the sweeping de-
of Dr. Smith, that " changes in the fluids can only be second in the series
0e rn?i'bid events: they can never hold the first place in that series: they can
r v'er be antecedents or first causes, but merely sequents or effects."* We
t jler think that the evidence existing on the subject, if failly examined, points
the blood as the seat of the primary operations of the morbific agent, and the
. Je?t of the changes which it is a part of its condition necessarily to produce.
Q regarding all derangements of the functions occurring in fever as the con-
theenCeS t^le mo'ecu'ar changes in that fluid, we proceed to examine into
e order in which these consecutive phenomena occur?the mode of their pro-
ton, and their mutual dependence one upon another.
e(j .uPP0sing Dr. Smith's to be a fair exposition of the views generally entertain-
th 1? country we find, upon reverting to his chapter on the theory of fever,
v0 doctrine maintained is, that subsequently to the supposed primary ner-
s 'Depression, " the organs the next to suffer are those of the circulation, then
?se of respiration ; and ultimately those which belong to secretion and excre-
o The condition of the nervous system which produces this derangement in
is circle of organs, occasions further in that portion of the circulating system,
t ?h consists of the capillary bloodvessels, that peculiar state which consti-
es inflammation : hence inflammation is almost always established in one or
th?fe the organs comprehended in the febrile circle and sometimes in all of
em." if thjs passage admits of a precise construction it must be that in con-
ecluence of a certain impression on the nervous system a state of general in-
clination exists (or at least a state approaching to inflammation) in the
J*P'.Uaries of all the organs, and which is equally likely to become actual inflam-
infltl0n 'n an*7 ?*" t^em during fever. Without denying this frequency of visceral
animation in fever, or the great necessity of recognising and combatting it, it
o ay be reasonably doubted if so variable and non-essential an occurrence?with
e exception?or one so dependent for its existence and its seat upon accidental
thUSeS~~as season> atmosphere, epidemic influences, states of constitution, &c.?as
, ese local inflammations are, can be properly admitted into the discussion of a
e?ry of fever. Either this combination of inflammation in some organ, with a
ij?CuHar state of the nervous system, is necessary to constitute fever, or it is not.
at it is, seems unlikely, since morbid anatomy fails to detect it in a large pro-
* On Fever, p. 330.
54 Extua-Limitks. [J11^ '
portion of instances. If it be not then according to this theory, nothing ^
remain but a certain peculiar affection of the nervous system to account
the phenomena of fever. But in the kind of fever under consideration
a local affection generally regarded as inflammatory, and which is so constan .
present and found to exercise so great an influence on the disease as to have d
considered by some eminent pathologists to be the essential cause of tyP. j
fever. It is that affection of the mucous glands of the small intestines descri
under the name of Dothin-enteritis. This affection claims a special consider
tion, since no one can impartially examine the evidence put forward in ^PP'L
of their views of the pathology of fever by Louis and Chomel, in France, or
cases incidentally published in the writings of Bright, Tweedie, Smith, Grav
and Stokes, Hodgkin, &c. in this country, without being convinced that in son
forms of fever?which farther examination will shew to be paludal fever?;' ^
dothin-enterite is a constant and a most important complication, if indeed it
not the pathological cause of the disease.
But if we seek an explanation of its occurrence in Dr. Smith's theory, v,e a,j
first at a loss to know why an impression on the brain and spinal chord sho" ^
lead to consequent inflammation in a part the most remote of any from their ^
fluence, and whose functions are under the control of a different portion of t
nervous system. Then, in very many cases, we find that the symptoms of }D
testinal irritation preceded this nervous impression?that in others, in w ,
death took place at an early period of fever, it was found far advanced?as ;
Louis so early as the 8th day?and could not therefore be regarded as a secoc
dary phenomenon. And that in other cases, in which the poison was present
in so concentrated a form that death took place before fever could be establish^ '
the glands of Peyer exhibited the same appearance as in that disease. The 1?
lowing interesting case of this kind is given by Dr. Christison.
In August last, twenty-two boys, living at a boarding-school at Clapha. '
were seized in the course of three or four hours with alarming symptoms of vl?
lent irritation in the stomach and bowels, subsultus of the muscles of the ar?Sj
and excessive prostration of strength. Another had been similarly attack
three days before. This child died in twenty-five, and one of the others'^
twenty-three hours. On examination after death, the Peyerian glands of the >n*
testines were found in the former case enlarged, and, as it were, tubercular" '
in the other, there were also ulcers of the mucous coat of the small intestine5'
and softening of that coat in the colon. A suspicion of poisoning having natural)
arisen, the various utensils and articles of food used by the family were examine '
but without success. And the only circumstance which appeared to explain t
accident was, that two days before the first child took ill, a foul cesspool na,
been opened, and the materials diffused over a garden adjoining the children5
play-ground. This was considered a sufficient cause of the disease, by V ^
Spurgin and Messrs. Angus and Saunders of Clapham, as well as by Drs. ^
tham and Chambers and Mr. Pearson, of London, who personally examined tn
whole particulars." There cannot, we think, be a doubt that their opinion >v
correct, and that nothing but the rapid termination prevented the developme
of the phenomena of fever in these cases; but in fairness to Dr. Christison*
should be added, that he considers " this opinion cannot be received with con
dence by the medical jurist and the physician, since it is not supported by an)
previous account of the effects of sulphuretted hydrogen." Perhaps these case,
may receive some confirmation from the following report (certainly not a iu
one) of a similar accident by Dr. Ai nott.* " In a mews behind Bedford-squar^
a stable had been let for a time to a butcher, and a heap of dung had bee^,
formed at the door, containing pigs' offal, pigeons' dung, &c. During the act 0
Fourth Report of Poor Law Commissioners, p 106.
On the Poison of Fever. 55
stabT'n? this heap, a coachman's wife and her three children, of an adjoining
ablc e' Eat ^?r a t'me at an ?Pen window nearly over the place until the insuffer-
befC stench drove them away : two of the poor children died of the poison
j?re36 hours, and the mother and other child narrowly escaped."
e)( ? Christison's cases the description of the appearances of Peyer's glands
*itJ ^ corresponds (especially the first case) with Dr. Bright's description, and
^vith u rePresentation given in one of the plates in his great work. As also
f i ?6 m'nute investigations of Dr. Staberoh, who regards the first stage of
tlie ''cular affection, as "an infiltration into the mucous coat, and especially
set. P.ts called Peyerian glands," hut also taking place, as he has repeatedly
filar 'n (''^ercnt parts of the colon, " and to which he considers the inflam-
ion of the mucous membrane secondary."?(Dublin Journal, Vol. 13.)
(] u*- farther?if dothin-enterite were a consequence of disordered circulation
t0 .g upon an impression on the brain and spinal chord, we might expect
fey"** '*? 'n other cases in which these organs are engaged, as in the periodic
fia6rS ant^ 'n tophus : but we do not, nor can any other local inflammation be
rile ? aS similarly constant in these diseases, and filling its place in the " feb-
p Clrcle." the contrary, M'Cartnev, Armstrong, and others, have fully
<*in ^at ^le vascu'ar congestion commonly found in these diseases is not of
'fifiammatory nature, and that, though it may remotely give rise in certain
,s<?s to an inflammatory re-action for its removal, it is yet a distinct patholo-
6'cal condition.*
ft?v W? ?^er opinions may be entertained of the relation of dothin-enteritis to
t er- One, that it is the primary cause : that fever is the sum of the symp-
of this inflammation?the other, that it is the specific effect of the septic
dis'SOn ^10m which typhoid fever originates, and like the other symptoms of this
ease, merely a link in the chain of sequences constituting fever.
erliaps the strongest arguments for the first of these opinions are the large
jt ?P?rtion of cases of typhoid fever in which it is found to exist,?the influence
r exercises upon the severity of the disease, and the effects of antiphlogistic
^'edies, more especially of topical blood-letting. It will be presently seen that
two first circumstances are equally well explained upon the second opinion.
Co' . reference to the effects of blood-letting it must be admitted that a very
isiderable amelioration of symptoms, and not unfrequently their total re-
Va'> has followed timely and free abstraction of blood, especially by leeches
iti k ?Ver a^"ecte(i intestine, and not only are the tenderness and pain
the part, with the meteorism and diarrhoea thus relieved, but the head-ache,
11 st, pulse, and other general symptoms commonly undergo at least a tempo-
. ry and partial improvement. But it may be doubted whether this is to be at-
' uted so much to the removal of inflammation as to an impression made upon
e general disease, by the new movement given to the circulation in general by the
tallest local abstraction of blood, and which is felt in every part of the system.
tJne or f-vvo circumstances may be cited in proof of this dynamic effect of
ceding, and to illustrate its application to the present case?1st, the well-known
^ct that the impression made upon the central organ of the circulation by the
ending from a few leech-bites is totally disproportionate to the quantity taken
wha>7- 2nd. The effect of leeching, or bleeding in some other disorders in
^ have known the disorder of several months' standing removed by thes^ppli-
^tion of a few leeches to the inguinal region before the leeches wer^
Removed. In ague also, an effect almost equally marked may
Ced by the same means. The following short case !* 'iabour-
*t- 20, was admitted into the Navan Hospital on the 22nd of February,
no supposition of inflammation could exist. In amenorrhoea, for instance,
I'or Dr. M'Cartnev's observations, see Dub. Med. Trans. Vol. 2, p. 574.
5G Extra-Limites. ? [Ju^- '
ing under tertian ague. The fit comes on two hours earlier at each period. Has
some tenderness on pressure, and fulness of epigastrium, thirst, tongue re
tip and edges?previous to entering hospital took two emetics and an apene
A fit took place about 3 a. m. on the 21st, and might be expected to recur at o
a. m. on the 23rd. I ordered 12 leeches to be applied on the evening of the ?
to the epigastrium, and a draught to be taken at bed-time, containing 20 dr?I
of laudanum. The fit occurred at 5 a. m. 4 hours later than it was expects ?
On the 25th it came on at 7, and the leeching being repeated on the evening of
26th, he sweated copiously during the night, and had no return of the fit. J*1?
was a slight return of it five days after, but from this time he got rapidly w '?
Without entering farther into the discussion of the theory, that dothinentef'
is the cause of the febrile phenomena, we pass on to submit certain considerate
in support of the view which regards it as an effect not of the fever or of the sta
of the nervous and circulating systems produced by the fever, but as the djreC.
effect of the poison itself?as one (probably the first) of the links in the chain 0
sequences, constituting fever, and one upon the occurrence of which soi?e 0
the others may probably depend. This view approaches nearer to that of Lou>9j,
who is of opinion that the affection of the follicles occurs in the beginning 0
the disease, than to that of Chomel, who seems inclined to admit its classify"
tion among the secondary inflammations, but differs from the definition of y1
former eminent pathologist, inasmuch as it seeks to establish the agency ot
morbid poison as the cause of fever, in place of his decision " that the cause ?
unknown." The following extract from his summary of the diagnostic symptom
of typhoid fever is important, as containing two particulars which we shall fan
to have a bearing upon this inquiry.
" Maladie aigue accompagnee d'un mouvement febrile plus ou moins intense'
variable dans sa duree; propre aux jeunes sujets, principalement a ceux qu*se
trouvent depuis peu de temps au mileau de circonstances nouvelles pour eux, dont
cause est inconnue; debutant par un frisson violent, l'anorexia, la soif, et dan3
la tres grande majorite des cas par des coliques et la diarrhee," &c. ..
The two circumstances here mentioned by Louis, of the subjects of typh?'
fever being those newly exposed to influences, the nature of which he conclude
are not known?and the diarrhoea which ushers in the complaint,?we conceiv-e
tend to support the theory that the intestinal affection is a consequence
of the
effort made by the excreting organs?more especially the liver?to rid the s)'s"
tem of the poison which has been introduced into the blood. It is easy toC?D"
ceive that the native of Paris, born and brought up in the atmosphere ol 'ts
fosses, and drinking all his life the tainted waters of its wells and river, i113^
habitually eliminate from the blood such products as are thus taken in unfit f?
assimilation or nutriment, and that to a constitution unused to such a task tne
consequence of taking into the circulation the same decomposing substance5
should be different. And, if we aid our conception of this fact by a referencet0
what takes place in the different classes of persons exposed to malaria of other
kinds in temperate climates, we shall see why intestinal affections should be
among the first consequences of the process; for it will appear that the liver lS
the organ by means of whose excretions the poison is attempted to be got rid 0 >
and according to the facility with which this is performed or otherwise, will b?
the chances of escape or the contrary, from the effects of the poison. It is
known that, while the lungs of a native of a warm climate are liable to becoWe
diseased upon removal to a cold one, the liver is the organ prone to suffer up?n
the inhabitant of a cold removing to a warm climate. It is also found that the
stranger from a colder country will rapidly contract fever from exposure to H13'
laria in a temperate climate, while the person newly arrived from a warm ??e
will not be similarly affected until that change in the order of his function5
termed acclimatization has taken place, and he becomes assimilated in habits to
the inhabitant of the same latitude.
(To be concluded in our next.)

				

